# DNA methylation regulators-related molecular patterns and tumor immune landscape in hepatocellular carcinoma

**DOI:** 10.3389/fonc.2022.877817

**Published:** 2022-08-26

**Authors:** Dingli Song, Zhenyu Zhou, Jie Wu, Tao Wei, Guang Zhao, Hong Ren, Boxiang Zhang

**Affiliations:** ^1^ Department of Thoracic Surgery, The First Affiliated Hospital of Xi’an Jiaotong University, Xi’an, China; ^2^ Department of Hepatobiliary Surgery, Sun Yat-Sen Memorial Hospital, Sun Yat-Sen University, Guangzhou, China; ^3^ Department of Hepatobiliary and Pancreatic Surgery, School of Medicine, The First Affiliated Hospital of Zhejiang University, Hangzhou, China

**Keywords:** hepatocellular cancer, DNA methylation regulators, classification, immune infiltration, signature

## Abstract

Increasing evidence showed that the dysregulation of DNA methylation regulators is a decisive feature of almost all cancer types and affects tumor progressions. However, few studies focused on the underlying influences of DNA methylation regulators-related genes (DMRegs) in immune cell-infiltration characteristics, tumor microenvironment (TME) and immunotherapy in HCC patients. In our study, the alterations of DNA methylation regulators modification patterns (DMRPs) were clustered from hepatocellular carcinoma (HCC) samples based on the expression of DNA methylation regulators as well as genetic and transcriptional features. In addition, based on molecular identification of three distinct molecular subtypes, we found that different DMRPs alterations were related to different clinicopathological characteristics, prognosis, and immune cells infiltration features. Moreover, we constructed and validated a DNA methylation regulators-related genes score (DMRegs_score) to predict the survival of HCC patients. A high DMRegs _score, which was characterized by more TP53 wild mutation, high expression of PD-1, CTLA-4, and remarkable immunity activation, was indicative of poor prognosis. Furthermore, we validated the expression of eight genes which were used for the prognostic signature in this risk score by RT-qPCR using tissues from our center. More importantly, DMRegs_score was highly correlated with targeted drug sensitivity. Additionally, we developed a highly accurate scoring system that could be used to improve the clinical applicability of DMRegs _score. In conclusion, these findings may contribute to a better understanding of DNA methylation regulators and provide new strategies for evaluating prognosis and developing more effective combination therapy for HCC patients.

## Introduction

Liver cancer, more specifically hepatocellular carcinoma (HCC), is the main leading cause of cancer-related death worldwide in 2020 (1, 2). At present, surgery is still the most effective treatment for HCC. However, due to the occult onset and rapid progress of HCC, patients often have lost the best opportunity for surgical treatment at the time of diagnosis. What’s more, the patients with HCC have poor prognosis because of high metastasis and recurrence rate (3). Therefore, exploring the molecular mechanism of HCC development and finding new early diagnosis and treatment targets are the focus of HCC research.

Epigenetic modifications, such as DNA methylation, play a crucial role in altering gene expression and contributing to disease development in mammals (4). According to present reports, methylation of the fifth carbon of the DNA cytosine within CpG dinucleotides is the most mechanistically understood form of DNA methylation (5). DNA methylation modification is a dynamic and vary process which is modulated by DNA methylation regulators, including DNA methyltransferases, DNA demethylases and DNA binding proteins (6–8). In addition, a growing body of evidence had demonstrated that dysregulation of DNA methylation regulators is a hallmark of almost all cancer types and affects tumor microenvironment (TME) or immunotherapy (7, 9, 10).

Co-inhibitory receptors Cytotoxic T-lymphocyte antigen 4 (CTLA4), programmed cell death protein 1 (PD-1), and programmed cell death ligand 1 (PD-L1) is expressed in the tumor microenvironment. Immunotherapy such as immune-checkpoint inhibitors (ICIs) that target these biomarkers activated the properties of effector T cells which can be able to kill cancer cells. Importantly, ICIs have radically reversed cancer therapy (11). Cancer immunotherapy targeting CTLA4, PD-L1, or PD-1 has become a widely used method of treating various types of cancer (12–14). Recently, anti-CTLA-4 was reported a survival benefit of HCC patients with sorafenib resistance (15). However, these immunotherapies were responding differently with patient to patient, and less than 20% of immune checkpoint blockade therapy was effective (16–18). It has been reported that the expression of PD-L1 and the status of tumor mutation burden (TMB) may be used as biomarkers to assess the effectiveness of immunotherapy (19–21). Interestingly, a recent study has revealed the characteristics of DNA methylation modification patterns of gastric cancer and explored the link between TME and DNA methylation modification, which indicated that DNA methylation may be a new predictor for immunotherapy (8). Moreover, DNA methylation regulators distinguish early HCC stages from chronic liver hepatitis B and C as well as healthy controls, intensify as the disease progresses, and is highly enriched in immune function-related genes such as PD-1 (22). These all suggest that DNA methylation regulators are closely related to immunotherapy and maybe predict the response to immunotherapy. However, it is still unclear how DNA methylation regulators affect tumors, especially cancer immunotherapy in HCC. Therefore, further elucidation of DNA methylation regulators could provide an attractive perspective on cancer immunotherapy.

In this study, we integrated patients from TCGA-LIHC cohort and ICGC LIRI-JP cohort to comprehensively evaluate the correlation between the DNA methylation modification patterns (DMRPs) and tumor immune landscape. First, we explored the expression of 20 DNA methylation regulators between normal and carcinoma tissues, and then identified 3 distinct DMRPs which were tightly correlated with immune cells infiltration and prognosis. In addition, we investigated the functional annotation to distinguish cancer associated signaling pathways to the three patterns. Moreover, we continued to quantify the DMRPs of individual HCC patients and assessed the clinical responses to immunotherapy based on DNA methylation regulators-related genes score (DMRegs_score). In conclusion, our novel DMRegs_score provides a reliable insight by which to identify and feature immune landscape of HCC, and the results suggest the DMRegs_score may be a biomarker for survival and precision treatment.

## Materials and method

### Data collection and preprocessing of public database

HCC patients with RNA-seq, genetic mutations (VarScan) and clinical information (included age, sex, TNM stage, follow-up time, and survival status) were obtained from the Cancer Genome Atlas (TCGA) data portal (TCGA-LIHC cohort, n=374) (http://portal.gdc.cancer.gov/). The copy number variant profiles (CNV) were downloaded from the UCSC xena (http://xenabrowser.net). The normalized data from another HCC cohort were downloaded from the International Cancer Genome Consortium (ICGC) database (ICGC LIRI-JP, n=231) (http://daco.icgc.org). GSE76427 (n=167) array was downloaded from Gene Expression Omnibus (GEO) database (https://www.ncbi.nlm.nih.gov/geo/). The 20 DNA methylation regulators were extracted from previous study (8). The TCGA and ICGC RNA sequence data (fragments per kilobase million, FPKM value) were transformed into TPM (transcripts per kilobase million) format. We excluded patients without complete clinical information and the survival time of 0, thus, a total of 685 HCC patients were further analyzed in this study. These detailed clinical information about 685 patients with HCC was presented in **Tables S1, S2**.

### Tissue samples and real-time PCR and immunohistochemical staining

Forty-one pairs HCC and nearby non-tumor tissues were collected from HCC patients who underwent hepatic resection in Sun Yat-Sen Memorial hospital between Nov 2020 and Mar 2021. Liquid nitrogen was used to store these samples until further analysis could be completed. The patients’ clinical data were also collected (**Table S3**). The study protocol was approved by the Ethics Committee of Sun Yat-Sen Memorial hospital and informed consent was obtained from each patient. We extracted the RNA from the tissues with Trizol (Takara, China), and performed reverse transcription using Prime Script RTase (Takara, China), according to the manufacturer’s protocol, respectively. According to the manufacturer’s instructions, real-time PCR was used to measure mRNA expression levels using SYBR green (Takara, China). A list of the primers used for real-time PCR is provided for **Table S4**. Immunohistochemical (IHC) staining was performed as described previously (23) using the following antibodies: Anti- CDCA3, Anti-CDC20, Anti-YWHAQ, Anti-ADH4, Anti-TRNP1, Anti-CYP2C9, Anti-CALU, Anti-APOC1. All antibodies used in the study are shown in **Table S5**. Quantitative evaluation of protein expression of IHC tissues was measured by ImageJ software. The number of stained cells was identified by trainable Weka segmentation.

### Interaction among DNA methylation regulators, copy number variant (CNV) analysis and gene mutation analysis

The crosstalk network diagram of multiple DNA methylation regulators was constructed by using “igraph” package, and presented the categories “Writers”, “Erasers” and “Readers” of these genes. The “RCircos” R package was used to visualize the location of 20 DNA methylation regulators in human chromosomes and the gain or loss status of copy number. The “maftools” R package was applied to evaluate the mutation status of 20 DNA methylation regulators and drawn the waterfall plots in HCC.

### Molecular subgroups-based clustering analysis for DNA methylation regulators

We performed the consensus clustering with Euclidean squared distance metric and the K-means clustering algorithm to identify distinct DMRPs based on the expression of 20 DNA methylation regulators by using the “ConsensusClusterPlus” R package. HCC samples were classified into k clusters with k=2 to k=9. Based on the consistent cumulative distribution function (CDF) and delta region graphs, an optimal number of clusters was determined (24). What’s more, we compared the relationships between molecular subgroups, clinical characteristics, and prognosis using the “survival” and “survminer” R packages. The clinicopathologies including age, gender, TNM stage.

### Function annotation based on gene set variant analysis (GSVA)

To identify the difference between the biological process of DMRPs, the “GSVA” package in R was utilized to performe GSVA enrichment analysis. GSVA, a nonparametric and unsupervised algorithm, can quantify the gene enrichment results in the sample of a gene expression dataset (25). In addition, we employed the “limma” R package to screen the significant variance in KEGG pathways and Hallmark pathways. The well-defined gene sets of “h.all.v7.4.symbols”, and “c2.cp.kegg.v7.2.symbols” were downloaded from MSigDB database.

### Identification of differentially expressed genes (DEGs) between DNA methylation regulators modification patterns

The previous consensus clustering analysis had classified patients into three distinct DMRPs based on 20 DNA methylation regulators, and we identified DNA methylation regulators modification-related differentially expressed genes (DMRegs) among different DMRPs. The Bayesian method of “limma” package was used to statistical analysis, and “venndiagram” R package was applied to visualize the DMRegs. The DMRegs with adjusted p<0.05 and |logFC|=0.5 were considered as screening criterion. To explore the potential functions of DMRegs, the “clusterprofler” package in R software was utilized for Gene Ontology (GO) and Kyoto Encyclopedia of Genes and Genomes (KEGG) enrichment analysis with adjusted P values < 0.05. What’s more, we further explored the gene cluster based on the expression profiles of DMRegs using unsupervised clustering methods.

### Construction of the DNA methylation regulators-related gene signature

We constructed a set of scoring system to quantify the DMRPs of individual patient with HCC by using the method of LASSO cox regression, and we termed the score as DMRegs_score. The DMRegs_score was developed as follows. Univariate cox regression analysis was performed to identify overlapping DMRegs related to survival with P-values <0.05. Then “glmnet” R package was employed to establish the DMRegs_score based on the expression of significant prognosis of DMRegs among gene clusters. Finally, the DMRegs_score was defined using a formula method like previous study: DMRegs_score = Σ (Expi * coefi), where Coefi and Expi represented the risk coefficient and expression of each gene, respectively. The Kaplan-Meier survival curve, the area under the curve (AUC) of the time-dependent receiver operating characteristics (ROC) curve were implemented to evaluate the predictive ability of the risk model. Combining clinical data with univariate and multivariate cox analysis were done to determine if the risk score was an independent feature.

### Estimation the relationship between DMRegs_score and TME, PD-1, PD-L1, and CTLA4

The ESTIMATE algorithm was used to calculate the tumor microenvironment (TME) scores, including stromal scores, immune scores, and estimate scores, which represented the infiltration of immune cells and stromal cells in TME (26). Moreover, based on the transcriptome profiles, we used “GSVA” R package to perform single sample gene set enrichment analysis (ssGSEA) to quantify the relative abundance of 23 immune cell types in the TME among different DMRPs (27). The marker genes of 23 immune cell types were acquired from a previous study, including activated B cell, MDSC, macrophage, regulatory T cell and so on (**Table S6**). Furthermore, we analyzed the relationship between the DMRegs_score and the expression of PD-1, PD-L1, CTLA4, and antigen presentation (HLA family).

### Characteristics of mutation burden and drug sensitivity analysis

By using “maftools” R package, the mutation annotation format (MAF) from the TCGA database was used to explore whether the mutations status of HCC patients was associated with high- or low-risk group. The tumor mutation burden score (TMB) was also calculated for each patient with HCC in both groups. Using the “pRRophetic” package, we analyzed the IC50 of several chemotherapeutic drugs which were commonly used to treat HCC in both groups.

### Validation the DMRegs_score and establishment of a nomogram assessing system

The reliability and predictive ability of this DMRegs_score was validated based on data from GEO dataset using same methods above mentioned. Furthermore, to expand the role of DMRegs_score in clinical practice, we used “rms” package to develop a nomogram predicting the prognosis of HCC patients, which combined the clinical features and DMRegs_score. The time-dependent ROC curves and calibration were performed to describe the predictive value of 1-, 2- and 3-year, respectively.

### Statistics analysis

The statistical analysis tools-R software (version 4.0.3, R Foundation for Statistical Computing, Vienna, Austria) was used in this study. Kruskal-Wallis tests or one-way ANOVA were used as nonparametric or parametric methods for comparisons of three groups, respectively using GraphPad Prism 8. And the results of RT-qPCR and IHC were conducted statistical analysis using pair t test. The forest plot and partial violin plots were generated by Sanger Box online tool. The hazard ratio (HR) and 95% confidence intervals (CI) were calculated. All statistical results with a P-value of <0.05 were considered significant.

## Results

### The landscape of DNA methylation regulators in HCC

**Figure 1:**
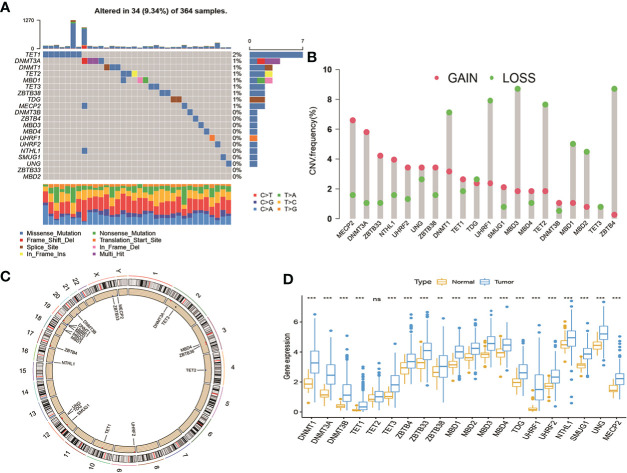
Landscape of genetic variations and transcriptional expression of DNA methylation regulators in HCC from TCGA cohort. **(A)** 34 of the 364 patients occurred genetic variations of 20 DNA methylation regulators with 9.34% mutation frequencies. **(B)** The frequency of CNV gain or loss among DNA methylation regulators. **(C)** The locations of CNV variations in DMRs on 23 chromosomes. **(D)** The expression level of 20 DNA methylation regulators between normal and HCC tissues. HCC, hepatocellular carcinoma; TCGA, The Cancer Genome Atlas; CNV, copy number variant.

A total of 20 DNA methylation regulators were identified in this study, including three writers (DNMT1, DNMT3A, and DNMT3B), three erasers (TET1, TET2 and TET3) and fourteen readers (MBD1, MBD2, MBD3, MBD4, ZBTB33, ZBTB38, ZBTB4, UHRF1, UHRF2, MECP2, UNG, TDG, NTHL1 and SMUG1). Based on a summary analysis of the incidence of somatic mutations in these 20 DNA methylation regulators, there was a low mutation rate for the patients with HCC from TCGA cohort (**Figure 1A**). Thirty-four patients can be found genetic mutation in available samples, and the mutation frequency of 20 DNA methylation regulators range from 1%-2%. The TET1 had the highest number of mutations of all the DNA methylation regulators (2%). The exploration of copy number variation (CNV) alteration frequency indicated common CNV alteration in the 20 DNA methylation regulators. DNMT1, UHRF1, TET2, MBD1/2/3 and ZBTB4 were focused on copy number deletion, while MECP2, DNMT3A, ZBTB33/38, NTHL1, UHRF2 and UNG had widespread frequency of CNV amplification (**Figure 1B**). In addition, the locations of the CNV alterations in the 20 DNA methylation regulators on chromosomes were presented in **Figure 1C**. The status of CNV alterations indicated that CNV might regulate the mRNA expression of DNA methylation regulators. Further analysis revealed that 19 out of 20 DNA methylation regulators were upregulated in tumor samples except TET2, although the expression of TET2 in tumor tissues is higher than in normal liver tissues (**Figure 1D**). These data suggest the 20 DNA methylation regulators may play important roles in HCC development.

### Identification of DNA methylation regulators-related modification patterns in HCC

**Figure 2:**
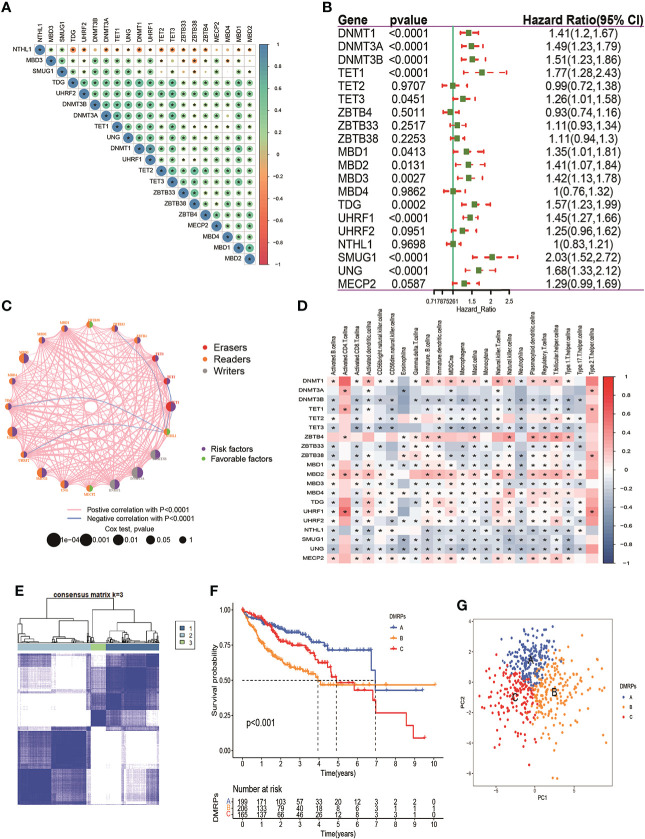
Prognosis of DNA methylation regulators and patterns of DNA methylation regulators modification. **(A)** The correlation of 20 DNA methylation regulators in HCC patients. **(B)** The prognosis of 20 DNA methylation regulators in HCC patients. **(C)** The interaction among 20 DNA methylation regulators in HCC. The pink and blue line represents positive and negative correlation. The size of the circle represents the p value of the log-rank test. Green points represent favorable factors for OS. Purple points represent risk factors for OS. **(D)** The correlation between 20 DNA methylation regulators and 23 types of immune cells. **(E)** The consensus cluster matrix for patients with HCC. **(F)** The survival analysis for different patterns of patients. **(G)** PCA analysis indicated significant separation among three patterns. * p < 0.05.

From above results, we speculated that DNA methylation regulators regulated deep-seated regulatory mechanism, which promotes us to further to investigate their potential functional in HCC. First, we gathered 570 patients from two HCC cohorts (TCGA-HCC and ICGC-LIRC) to explore the expression patterns of DNA methylation regulators involved in tumorigenesis. Spearman correlation analysis was utilized to assess mutual regulation among these DNA methylation regulators (**Figure 2A**). The results revealed MBD2, ZBTB33 and TET2 had a significant positive correlation with other DNA methylation regulators. Next, Cox regression and Kaplan-Meier analysis were performed to classify the prognostic relationship of these regulators with the HCC patients. Forest plot revealed that DNMT1/3A//3B, TET1/3, MBD1/2/3, TDG, UHRF1, SMUG1 and UNG were significantly associated with shorter overall survival and were considered as risk factors in HCC patients (**Figure 2B**). The crosstalk network showed the interaction and the prognostic value among 20 DNA methylation regulators in patients with HCC (**Figure 2C**). In addition, to better understand the role of DNA methylation regulators in tumor immunity, we explored the correlation between the 20 DNA methylation regulators and TME-infiltration immune cells using Spearman correlation analysis, and we found a significant negative relationship between most of these regulators and immune cells interaction (**Figure 2D**). Among them, DNMT1, ZBTB4 and MBD2 presented a strong positive correlation with most types of immune cells, such as activated CD4+ T cells, immature dendritic cells, and regulatory T cells. These results revealed that DNA methylation regulators regulated tumor microenvironment, which might provide strategies for immunotherapy. To understand the heterogeneity of DNA methylation regulators in HCC patients, we then performed unsupervised clustering analysis to classify patients based on the expression profiles of 20 DNA methylation regulators (**Figures S1A–I**). These results indicated that k=3 could achieve the best cluster efficacy. Therefore, the patients were categorized into three different DMRPs, including pattern A (n=199), pattern B (n=206) and pattern C (n=165) (**Figure 2E**). The Kaplan-Meier curves revealed that the pattern C had the poorer prognosis than pattern A and pattern B (**Figure 2F**). PCA analysis suggested three clusters were apparently discernible dimensions in the 20 DNA methylation regulators transcription profiles (**Figure 2G**).

### Distinct DMRPs and function pathways analysis

**Figure 3:**
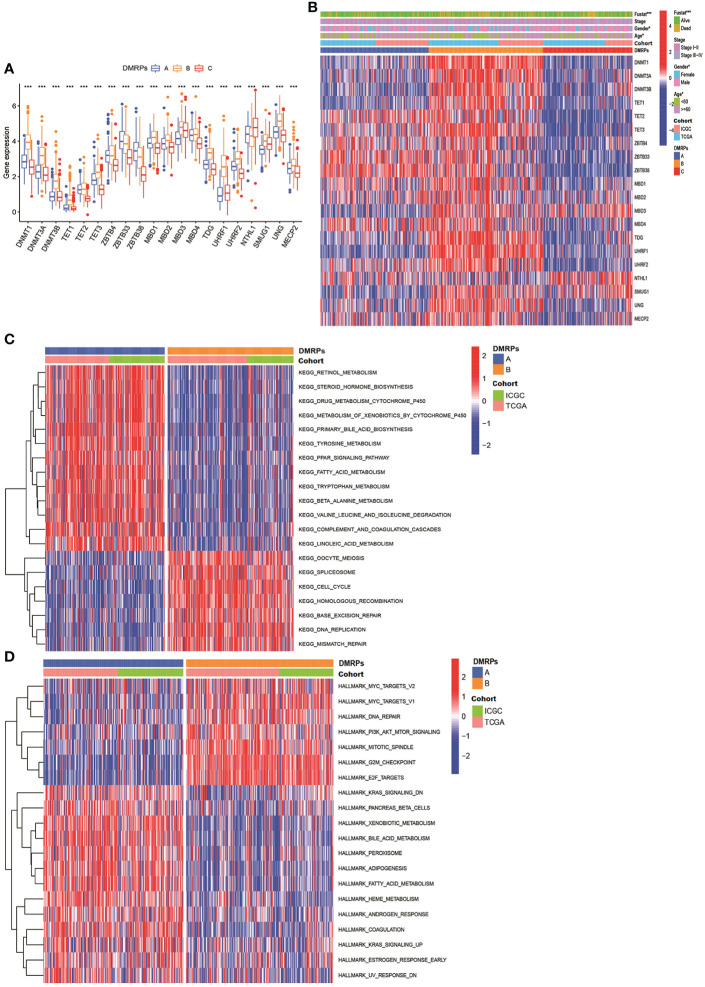
The clinical features of different DNA methylation regulator-related patterns and relevant function mechanism. **(A)** The expression of 20 DNA methylation regulators among three DNA methylation regulator-related patterns. **(B)** The heatmap of the differences between the expression of 20 DNA methylation regulators and clinicopathological factors. **(C, D)** GSVA showed the results of KEGG and Hallmark pathways in distinct DNA methylation regulators related modification patterns, respectively. * P < 0.05, ** P < 0.01, *** P < 0.001, **** P < 0.0001.

What’s more, we found most of these DNA methylation regulators were highly expressed in pattern B. Pattern A was presented high expression levels of ZBTB4/33/38. Only NTHL1 was relatively highly expressed in pattern C (**Figure 3A**). These data indicated that three DMRPs had distinct characteristics in the DNA methylation regulators modification. Furthermore, as shown in the heatmap, we explored the association between the various clinicopathological characteristics and three patterns based on 20 DNA methylation regulators expression of the metadata set (**Figure 3B**). And we found pattern B was related to female patients and patients younger than 60 years old (p<0.05), and the number of deaths were higher than the other two patterns (p<0.001). Hence, the patients in pattern B had poorer prognosis than other two patterns. The comprehensive comparisons of the clinical features of the three DMRPs suggested most of these DNA methylation regulators played potential roles on oncogenesis. To investigate the underlying molecular mechanism and signal pathways to each DMRP, the GSVA enrichment analysis based on KEGG and Hallmark gene sets were conducted. As presented in **Figures 3C, D**, the results indicated significant difference between three patterns. The pattern B was significantly enriched in cell cycle and cancer-associated pathways, including DNA replication, PI3K/AKT/mTOR signaling. The pattern A was highly enriched in processes of metabolism and some carcinogenic activation pathways, such as retinol metabolism, Wnt pathway, mTOR pathway, and TGF-β signaling pathway (**Figure 3C** and **Figures S2A, B**, **Table S7**). However, the pattern C mainly presented enrichment metabolism in tyrosine and drug (**Figures S2C, D**). Thus, our results identified each DMRP is associated with its specific clinicopathological features and signaling pathways. Some previous studies had reported that PI3K/mTOR signaling pathway played a critical regulatory role in the tumor microenvironment. In immunology, mTOR was becoming as a key regulator of immune responses, which played an essential regulatory role in the differentiation and function of both innate and adaptive immune cells (28).What’s more, the previous results revealed that most of DNA methylation regulators were associated with multiple immune cells. Consequently, it’s essential to explore the correlation between DMRPs and tumor immune cells infiltration.

### Characteristics of the TME immune cell infiltration in distinct DMRPs

**Figure 4:**
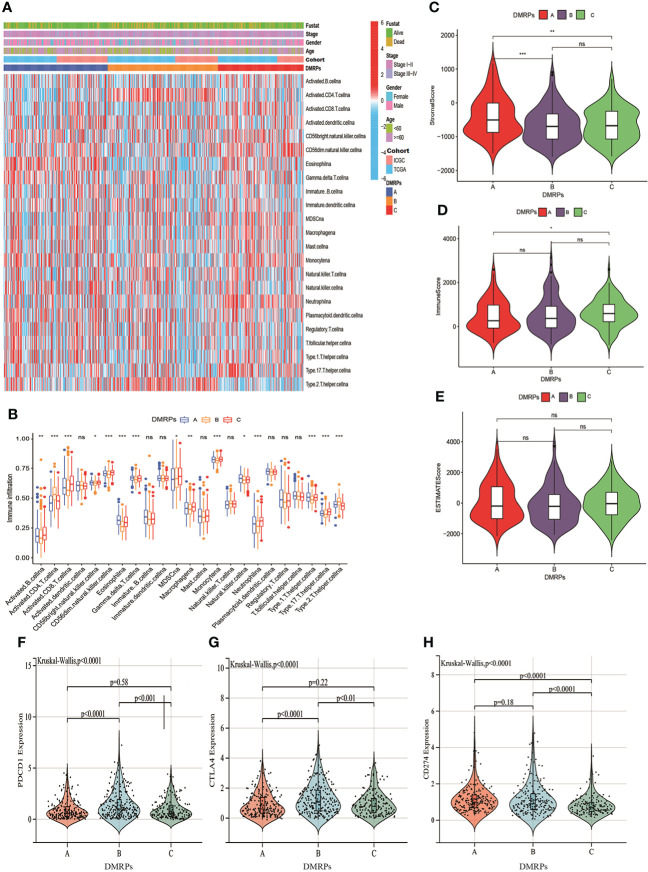
Characteristics of the TME immune cells infiltration in distinct DNA methylation regulators related modification patterns. **(A)** The heatmap of immune cell infiltration in three patterns. **(B)** The distribution of immune cells among three patterns. **(C–E)** The differences of TME score (stromal score, immune score and estimate score) among three patterns. **(F–H)** The expression levels of three important immune checkpoint in the three DMRPs. DMRPs: DNA methylation regulators related modification patterns; TME: tumor microenvironment. * P < 0.05, ** P < 0.01, *** P < 0.001, **** P < 0.0001. ns, no significance.

Previous studies had reported DNA methylation played a crucial role on tumor immune microenvironment (29, 30). Hence, we evaluated the relationship between three DMRPs and 23 types of immune cell subsets of every HCC sample using ssGSEA. The heatmap displayed significant differences in 23 immune cells infiltrations among these DMRPs with various clinicopathological features (**Figure 4A**). We found that natural killer T cells, eosinophils, gamma T cells and type 1T helper cells had a higher proportion in pattern A than pattern B and C. The infiltration level of activated B cells, activated CD8 T cells, CD56bright natural killer cells, CD56dim natural killer cells, MDSCs, macrophages, monocytes, neutrophils, and type 17 T helper cells were higher in pattern C, while activated CD4 T cells and type 2 T helper cells had significantly higher infiltration in pattern B (**Figure 4B**). The immune landscape stated clearly that the significant differences of the relative expression of multiple immune infiltration cells among three DMRPs. To explore the influence of DNA methylation regulators on the TME of HCC, further analysis of TME scores (immune score, stromal score and estimate score) were evaluated by using the ESTIMATE algorithm. These results showed that the stromal score was the highest in pattern A than other two patterns, but there was no statistical significance between pattern B and pattern C (**Figure 4C**), and the highest immune score was found in pattern C (**Figure 4D**). However, there weren’t any significant differences in estimate scores among the three patterns (**Figure 4E**). In addition, blocking therapy against immune checkpoints was believed to increase the aggressiveness of the host immune system against tumor cells. Hence, we further assessed the expression levels of PD-1, PD-L1 and CTLA4 among three DMRPs. The analysis of immune checkpoints suggested that pattern B exited the highest expression of PD-1compared to pattern A and pattern C (**Figure 4F**). Similarly, the pattern B had a higher expression level of CTLA4 than pattern A and pattern C (**Figure 4G**). We also compared the PD-L1 expression levels in different DMRPs and found a significant upregulation in pattern A (**Figure 4H**). Based on these results, we identified that HCC patients with specific DMRPs were associated with different immune infiltration characteristics, which might influence the development and progression of HCC. What’s more, potential immunotherapy could be selected according to the expression of immune checkpoints in patients with different DNA methylation regulators modification.

### DMRPs-related DEGs and gene clusters in HCC

**Figure 5:**
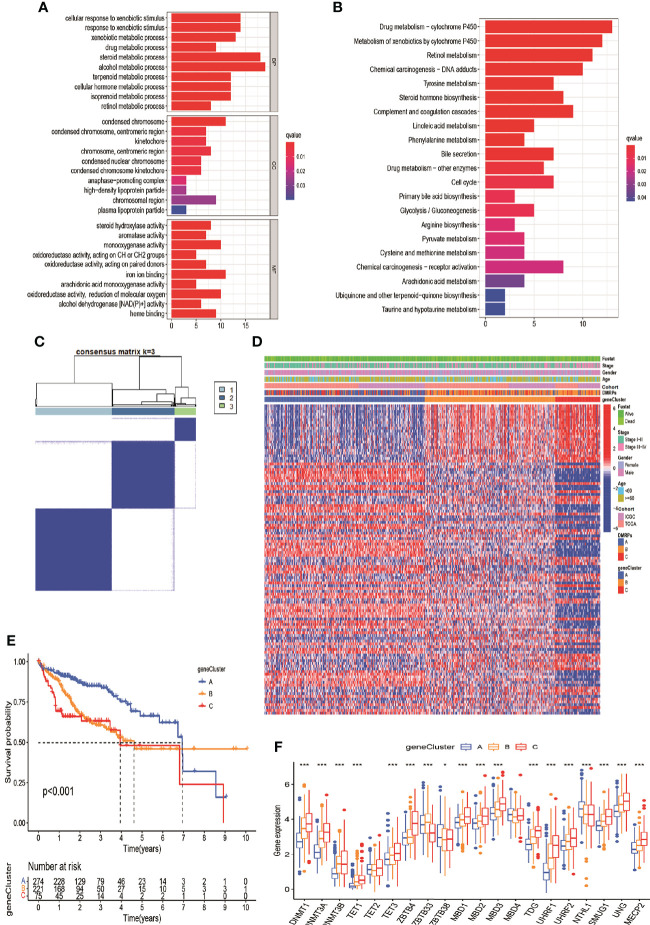
DNA modification pattern-related DEGs and gene clusters in HCC. **(A, B)** GO and KEGG enrichment analysis for DEGs of DMRPs. **(C)** Consensus cluster matrix of 570 patients for k = 3. **(D)** The relationship of clinical characteristics and unsupervised clustering of DEGs. **(E)** Kaplan-Meier curves of three gene clusters. **(F)** The expression levels of 20 DNA methylation regulators among three gene clusters. * P < 0.05, ** P < 0.01, *** P < 0.001, **** P < 0.0001.

To investigate the potential genetic alterations and expression perturbations affected by the three DMRPs in HCC, we screened a total of 151 DMRegs from three DMRPs using “limma” R package based on the metadata set (**Figure S3A**). Function annotation for these genes showed that some DMRegs were significantly correlated with metabolism in biological processes (**Figure 5A**), while material metabolism, glycolysis, and cell cycle were mainly pathways in KEGG analysis (**Figure 5B**). When we used univariate cox regression analysis to explore their relationship with the OS status of the HCC patients, and 112 DMRegs with significant prognostic value were selected to further identify (**Table S8**). Based on the expression profiles of these significant genes, we performed consensus clustering analysis and obtained three genomic clusters, namely gene Cluster A-C (**Figure 5C** and **Figures S3B–I**). The heatmap displayed the distinct characteristics of three phenotypes on the expression of prognostic DMRegs, and clinical analysis showed geneCluster C tended to relate to the advance TNM stage (**Figure 5D**). Additionally, the survival analysis demonstrated geneCluster C had a poorer survival rate (**Figure 5E**). Obviously, the expressions of DNA methylation regulators were significantly different among three gene clusters in the metadata set (**Figure 5F**). Most of these DNA methylation regulators (15/20) were presented higher expression levels in geneCluster C, such as DNMT1/3A/3B, TET1/3, ZBTB4, MBD1/2/3, TDG, UHRF1/2, SMUG1, UNG and MECP2. However, there was no significant difference between the expression of TET2, MBD4 and three gene clusters. Above all, these results illustrated the existence of specific clusters of genes in different DMRPs, which further supported the important roles of the three DMRPs in HCC.

### Generation of DMRegs_score in HCC

**Figure 6:**
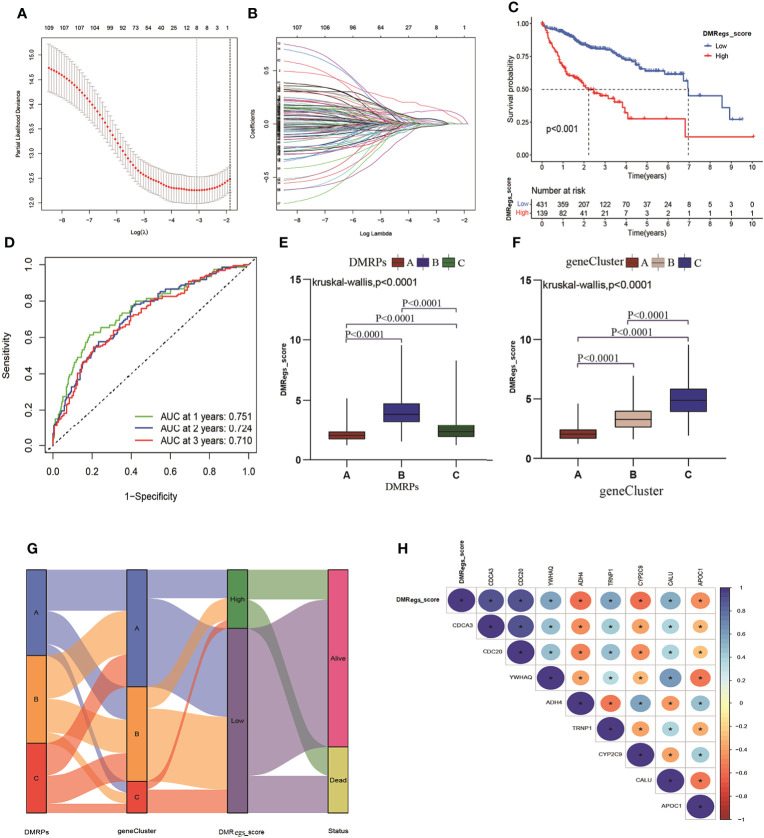
Generation of DMRegs_score. **(A, B)** The screen of candidate prognostic genes through LASSO cox regression analysis. **(C)** Survival analysis of the OS between different risk groups. **(D)** The predictive value of DMRs score. **(E, F)** The differences of DMRegs_score among DMRPs and gene clusters, respectively. **(G)** Sankey diagram of the DNA patterns, gene clusters, DMRegs_score and survival status. **(H)** The correlation between DMRegs and DMRegs_score. * P < 0.05, ** P < 0.01, *** P < 0.001, **** P < 0.0001.

In order to more understand the impact of these DNA methylation regulators on patients on HCC patients and better apply the research results to clinical practice, we constructed a DMRegs_score based on 112 prognostic DMRegs. As displayed in **Figures 6A, B**, we performed LASSO cox analysis to build prognostic risk score based on optimal λ. According to the results, we obtained 8 genes (CDCA3, CDC20, YWHAQ, ADH4, TRNP1, CYP2C9, CALU and APOC1) in the signature, including five high-risk genes (CDCA3, CDC20, YWHAQ, TRNP1, and CALU) and three low-risk genes (ADH4, CYP2C9, and APOC1) (**Figure S4A**). We therefore chose these 8 genes to establish the DMRegs_score using following: DMRegs_score = (0.1507*expression of CDCA3) + (0.1259*expression of CDC20) + (0.0408*expression of YWHAQ) + (0.0220*expression of TRNP1) + (0.0443 * expression of CALU) + (-0.0189*expression of CYP2C9) + (-0.0096 * expression of APOC1) + (-0.0055 * expression of ADH4). With an optimal survival cut-point value of 3.75, we divided the patients into high-risk group (n=139) and low-risk group (n=431) (**Table S9**). A significant worse prognosis was observed for the patients in the high-risk group compared to the low-risk group (**Figure 6C**). The differential expressions of eight genes between high- and low-risk group were presented in **Figures S4B–J**.

Three genes (ADH4, APOC1 and CYP2C9) were highly expressed in low-risk group, and the expression levels of five genes (CDCA3, CDC20, YWHAQ, TRNP1, and CALU) were higher in high-risk group. We also explored the relationship between eight genes expression and clinicopathological factors (**Figure S5A**). These results revealed advanced stage and death patients had higher expression level of high-risk genes. The mutated frequency of these genes was drawn using “maftool” package, and we found the few alterations happened in eight genes based on all HCC patients from TCGA (**Figure S5B**). The loop graph presented the chromosome locations and the gain or loss status of CNV among these risk genes (**Figures S5C, D**). The mutated frequency and CNV results indicated that these eight genes were epigenetically regulated by DNA methylation rather than DNA mutation or genomic alteration. What’s more, the 1-, 2-, and 3-year survival rate of DMRegs_score were illustrated by AUC values of ROC curves, 0.751, 0.724 and 0.710, respectively (**Figure 6D**). In addition, we discovered significant differences in DMRegs_score between three DMRPs and three gene clusters (**Figures 6E, F**). The DMRegs_score was highest in pattern B, while that of pattern A was lowest. Differently, the gene cluster C had the highest DMRegs_score than the other two phenotypes. The Sankey diagram showed the distribution of patients in three DMRPs, three gene clusters, DMRegs_score and survival status (**Figure 6G**). In addition, the correlation strength among these genes in DMRegs_score was presented in **Figure 6H**.

### Assessment of TME, immune-infiltrating cells and immune checkpoint between different risk group

**Figure 7:**
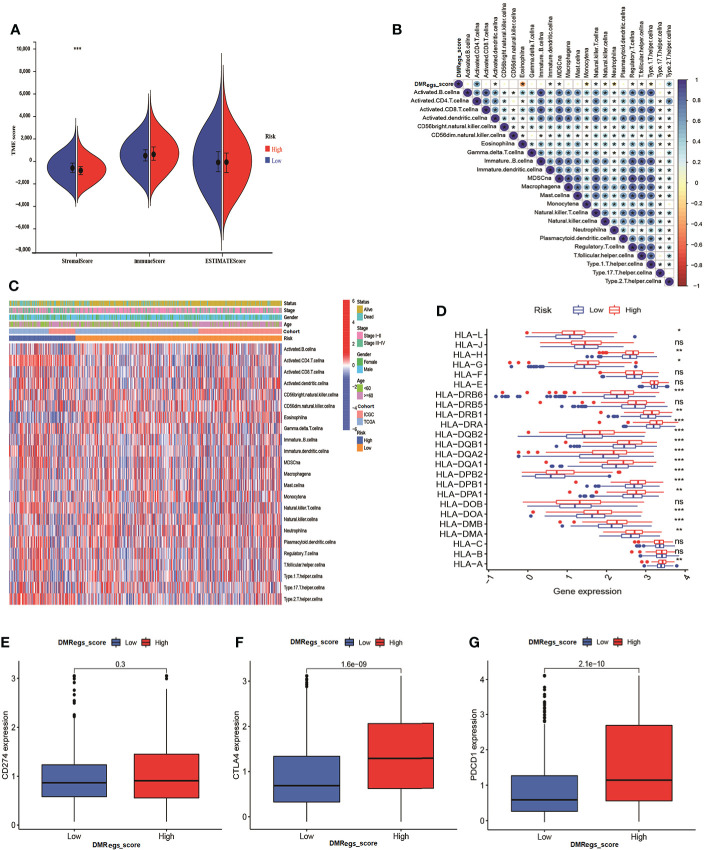
The immune cell infiltration characteristics of DMRegs_score in HCC patients. **(A)** The differences of TME score in the two risk groups. **(B)** The relationship of DMRegs_score and 23 types of immune cells infiltration. **(C)** The landscape of immune cells infiltration of DMRegs_score in patients with different clinicopathological features. **(D)** The expression differences of HLA-related genes between two risk group. **(E–G)** The expression level of CD274, CTLA4 and PDCD1 in two risk groups. * P < 0.05, ** P < 0.01, *** P < 0.001, **** P < 0.0001. ns, no significance.

In order to clearly understand the relationships between DMRegs_score, TME and immune infiltrating cells, firstly, we evaluated the differences of TME scores between high- and low-risk groups. **Figure 7A** illustrated the low-risk group had higher stromal score than high-risk group. However, no significant differences of immune score and estimate score were observed between two risk groups. As represented in **Figure 7B**, the correlation of DMRegs_score and 23 immune-infiltrating cells illustrated DMRegs_score was positively correlated with actively CD4 T cells, MDSC, immune dendritic cells, natural killer T cells and type 2 T helper cells, while was negatively correlated with eosinophil, monocytes, neutrophil, regulatory T cells, and type 1T helper cells. And the heatmap displayed the abundance of 23 types of immune cell infiltration in patients with different clinical features (**Figure 7C**). Notably, we found the DMRegs_score was associated with T cells, so we further explored the expression of human leukocyte antigen (HLA) related genes in different risk groups (**Figure 7D**). Most of the HLA-related genes presented higher expression level in high-risk group. Similarly, analysis of three important immune checkpoints revealed higher expression of PD-1 and CTLA4 in high -risk group, while the expression of PD-L1(CD274) between different risk groups was no significant difference (**Figures 7E–G**). These results suggested a strong correlation between DMRegs and TME of HCC patients. We guested that the DMRegs played crucial roles on the development of HCC through influenced the immune status of HCC patients.

### Evaluation of the relationship between DMRegs_score and clinical characteristics

**Figure 8:**
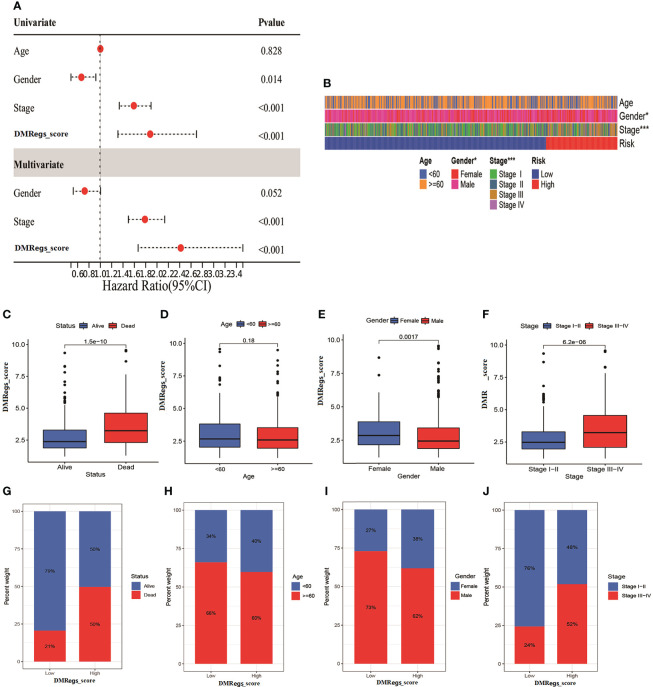
The clinical features of DMRegs_score in HCC patients. **(A)** The forest plot of univariate and multivariate cox analysis for independent prognostic factor. **(B)** The correlation heatmap of two risk groups with different clinical features. **(C–F)**. The correlation between DMRegs_score and survival status, age, gender and TNM stage. **(G–J)** The percentile of patients with different clinical features in two risk group. * P < 0.05, ** P < 0.01, *** P < 0.001, **** P < 0.0001.

To investigate the effect of the DMRegs_score on clinical characteristics, we performed univariate and multivariate cox regression to identify whether the DMRegs_score can be an independent predicator to predict the prognosis of HCC patients. The forest plot showed the DMRegs_score could function as an independent prognostic indicator for overall survival in the multivariate analysis (**Figure 8A**). The clinical heat map showed

more advanced stage patients gathered in high-risk group (**Figure 8B**). The DMRegs_score was significantly higher in death, female, and advanced stage patients, while no statistical difference was observed in age (<60 or >=60) (**Figures 8C–F**). What’s more, we also explored the proportion of patients with different clinical characteristics in two risk groups (**Figures 8G–J**). The Kaplan-Meier curves suggested that, whatever the age, gender and TNM stage, the patients in high-risk group had poorer survival rate than patients in low-risk group (**Figure S6**).

### Function enrichment, tumor mutation burden and drug sensitivity analysis

**Figure 9:**
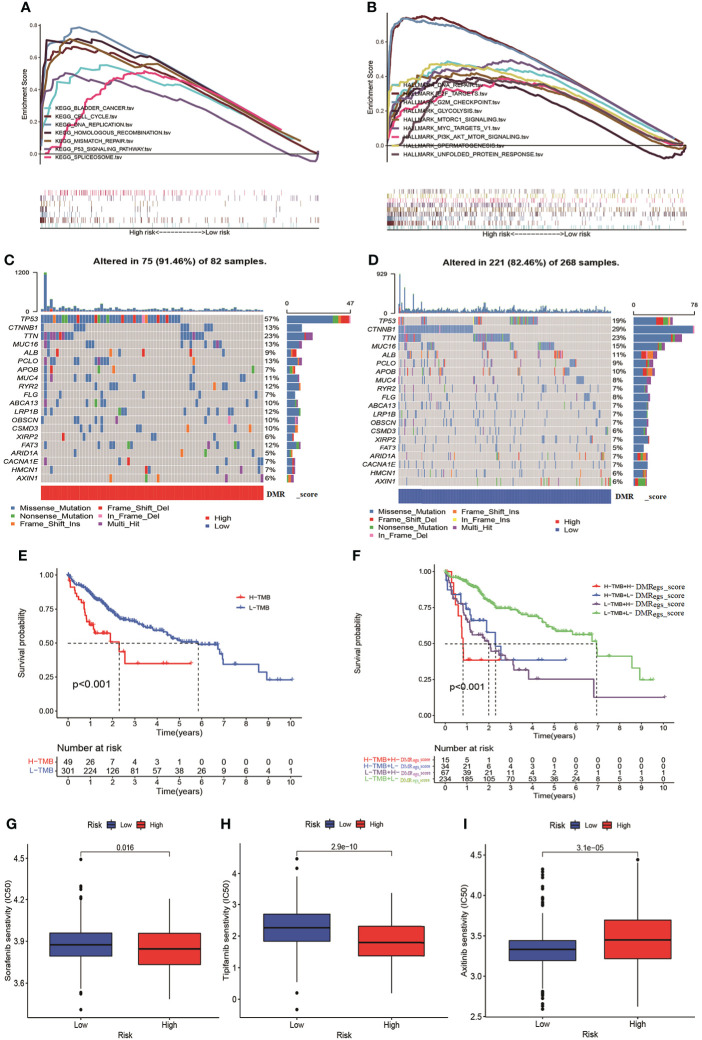
The TMB and drug sensitivity analysis for patients with different risk score. **(A, B)** The GSEA analysis in different risk groups. **(C, D)** The characteristics of tumor genetic alterations in two risk groups. **(E)** The survival analysis of patients with different TMB. **(F)** The survival analysis among four patient groups stratified by both TMB and DMRegs_score. (**G–I**) The drug sensitivity analysis of patients with different risk score. TMB: tumor mutation burden.

To evaluate the potential molecular mechanism of this signature, we applied GSEA based on KEGG and Hallmark gene set. The function enrichment analysis demonstrated that cell cycle, glycolysis and cancer-associated pathways were mainly enriched in high-risk group (**Figures 9A, B**). These results were consistent with previous DMRPs and gene-related phenotypes. In previous studies reported TMB played a crucial role in cancer progress and immunotherapy. Therefore, we also assessed the mutational feature between two risk groups based on TCGA-LIHC cohort. The high-risk group had a higher mutation frequency (91.46%) in 82 patients, while the alteration frequency in the low-risk group was 82.46% (**Figures 9C, D**). The top ten mutated genes in the high- risk group were TP53, CTNNB1, TTN, MUC16, LRP1B, PCLO, APOB, MUC4, RYR2, and FAT3, and the most common type of mutation was missense mutation. Moreover, the Kaplan-Meier curves showed that patients with lower level of TMB had a more favorable survival rate than high-TMB (**Figure 9E**). Combined TMB with the DMRegs_score to assess the prognosis of HCC patients, we found patients with both high-TMB and high DMRegs_score had the poorest prognosis (**Figure 9F**). We further screened target therapeutic drugs for the treatment of HCC patients to assess the sensitivity in different risk groups. Importantly, we observed that patients in high-risk group had lower IC50 value for sorafenib (VEGFR inhibitor), tipifarnib (farnesyltransferase inhibitor), A.443654 (AKT inhibitor), veliparib (PARP inhibitor), olaparib (PARP inhibitor), IPA-3 (PAK inhibitor), GSK-650394 (SGK inhibitor) and CCT018159 (Hsp90 inhibitor) (**Figures 9G, H**, **Figure S7**), while the IC50 values of axitinib (VEGFR inhibitor), motesanib (VEGFR), CCT007093 (PPM1D inhibitor), and lesteurtinib (JAK inhibitor) were higher in patients with high DMRegs_score (**Figure 9I**, **Figure S7**). In a word, DMRegs_score was significantly correlated with TMB and patients’ clinical response to targeted therapy.

### Verification the DMRegs_score and development a nomogram to predict prognosis

**Figure 10:**
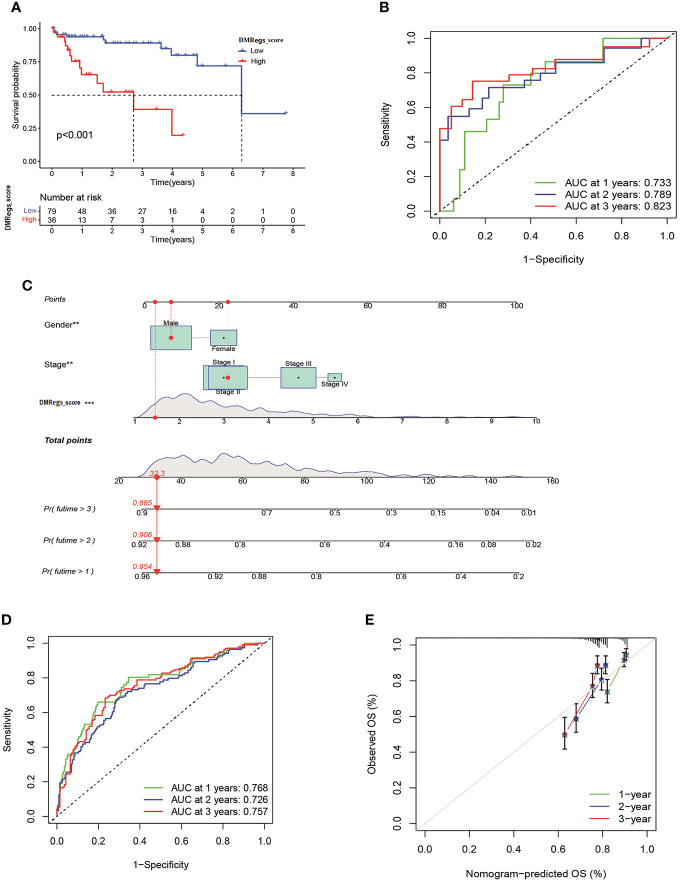
Validation of DMRegs_score in GSE76427 set and construction of nomogram. **(A)** The Kaplan- Meier curves of the OS between the two groups. **(B)** The predictive value of DMRegs_score was presented by ROC curves. **(C)** Nomogram to predict the 1-, 2- and 3-year OS of HCC patients in training set. **(D, E)** ROC and calibration curves of Nomogram for predicting of 1-, 2-, and 3-year OS in training set. ROC: receiver operating characteristic.

To validate the reliability of the DMRegs_score, we used GSE76427 as external validation group. Patients were categorized into high- and low-risk groups. The multivariate cox analysis revealed the DMRegs_score could be an independent prognostic factor (**Figure S8**). Survival analysis indicated the high DMRegs_score had bad survival rate (**Figure 10A**). And the ROC curves showed the DMRegs_score still had accurate AUC values, 1-year for 0.733, 2-year for 0.789 and 3-year for 0.823(**Figure 10B**). We further established a nomogram based on the risk data of DMRegs_score and the patients’ clinical features from metadata set. The nomogram composed of DMRegs_score, gender and TNM stage (**Figure 10C**). The AUC value of nomogram for 1-year, 2-year, and 3-year were 0.768,0.728, and 0.757, respectively (**Figure 10D**). In addition, we used calibration curves to confirm this nomogram prediction model (**Figure 10E**). However, compared with the AUC value of DMRegs_score, the nomogram scoring system had a slightly weaker predictive ability.

### Validation of the expression levels of the eight risk genes which are used for the prognostic signature

**Figure 11:**
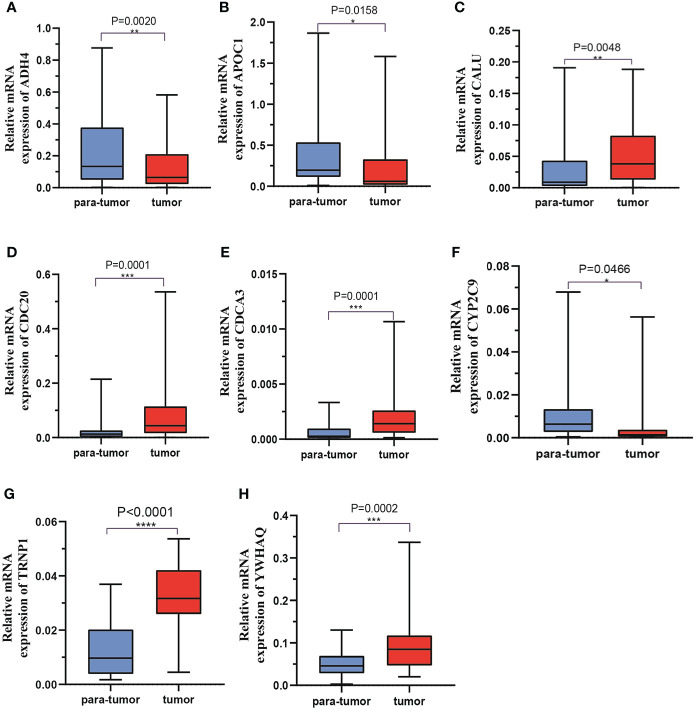
The mRNA expression levels of 8 DMRegs of prognostic signature in hepatocellular carcinoma tissues and corresponding normal tissues by RT-qPCR.

**Figure 12:**
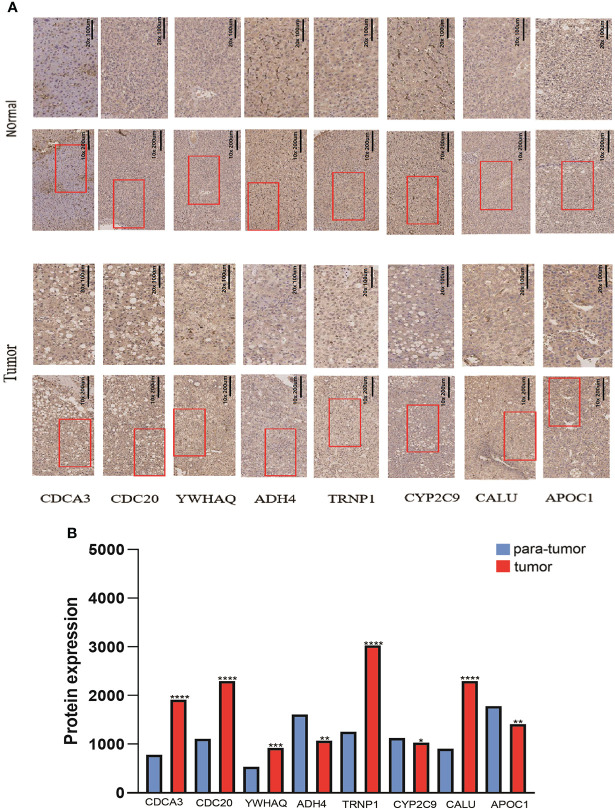
The protein expression levels of 8 DMRegs of prognostic signature in hepatocellular carcinoma tissues and corresponding normal tissues by IHC.

Forty-one HCC tissues and adjacent normal tissues were used to detect the mRNA and protein expression of eight genes in this risk score by qRT-PCR and IHC. As presented in **Figure 11**, the mRNA expression level of ADH4, APOC1, and CYP2C9 were downregulated while those of CALU, CDC20, CDC3A, TRNP1, and YWHAQ were elevated in HCC tissues compared to the levels in the paired normal tissues. The results of IHC staining showed the same result as qRT-PCR, and almost genes expressed in cytoplasmic except TRNP1 expressed in nucleus (**Figure 12**).

## Discussion

DNA methylation is closely related to carcinogenesis, tumor progression and metastasis (31, 32). In addition, by impacting multiple oncogenic pathways and tumor suppressor genes, DNA methylation regulators contribute to carcinogenesis in a broad range of tissue histologist (33, 34). In our study, based on expression levels of 20 DNA methylation regulators, we identified three DMRPs, and each DMRP correlated with different prognosis and signal pathway. Furthermore, Pattern B had the poorer prognosis and enriched in cell cycle and cancer-associated pathways, while the other two patterns mainly enriched in processes of metabolism. Besides, in order to confirm the efficacy of this regulatory mechanism, we applied consensus clustering analysis and found that genes cluster C was closely associated with more advanced TNM stage and poorer prognosis. Therefore, our study elucidates that the involvement of DNA methylation regulators in tumor development from a horizontal perspective, and provides new insights into the molecular networks involved in the regulation of DNA methylation regulators.

DNA methylation regulators can also impact the activation, differentiation, and functional fate of immune cells, which serve as a surveillance system against cancer (9, 35). For example, DNA demethylase TET2 promotes melanoma progression by maintaining the immunosuppressive function of myeloid cells and enhances anti-tumor immunity by governing G-MDSCs and CD8 + T-cell numbers (36, 37). The vast majority of studies, however, focused on a single DNA methylation regulator and its effect on altering TME (38–40). The immune cells infiltration characteristics, which are mediated by multiple synergistic DNA methylation regulator, have remained poorly understood. In this study, the DMRPs and TME immune infiltrating cells were closely related to each other. In addition, the immune scores and immune infiltrating cell types were significantly different in three DMRPs. More importantly, Based on DNA methylation regulators features found in individual tumors, we developed an effective DMRegs_score model and demonstrated its predictive ability. The clinicopathological features, prognosis and stromal score of high- and low-risk score were significantly distinct. Moreover, DMRegs_score was positively correlated with actively CD4+ T cells, MDSC, immune dendritic cells, natural killer T cells and type 2 T helper cells. Previous studies have indicated that more CD4+ T cells suggest a better prognosis (41, 42). However, our study found the tumor stage was more advanced in the population with high-risk group and more CD4+ T cells, and this group had better efficacy with drugs such as sorafenib and higher expression of PD-1 and CTLA-4, suggesting that the group may have a better outcome with combination therapy despite their late staging. In addition, other cancer immunotherapies in clinical trials, including dendritic cell vaccines and oncolytic viruses are also associated with TME immune infiltrating cells, such as T cells and dendritic cells (43, 44). During tumor growth, the stromal component is powerfully constricting to the immune cells, which can both be present in the tumor capsule and throughout the tumor tissue in order to prevent them from exerting anti-tumor effects (45). This is also supported by the evidence of strong stromal activation in DMRP C, where the activation of immune cells was inhibited by the AKT/mTOR pathway.

ICIs have been found to be effective in combinatorial strategies in advanced HCC patients (46). However, there is no effective biomarker for assessing the response to ICIs therapy and the prognosis of patients with HCC (47). Our detailed analyses indicated that the DMRegs_score signature probably is a robust and reliable biomarker to assess HCC patients’ responses to ICIs and TKIs. Patients with high DMRegs_score displayed higher expression of PD-1 and CTLA-4 compared with patients with lower DMRegs_score. Previous studies indicated that higher expression of PD-1, CTLA-4 and TMB might be inclined to respond to ICIs (48, 49). Thus, we concluded that patients with high DMRegs_score, which have high TMB, high expression of PD-1, and CTLA-4 might be more suitable to ICIs. Furthermore, for advanced hepatocellular carcinoma (HCC), tyrosine kinase inhibitors (TKIs) are effective therapeutic strategies. High DMRegs_score group had lower IC50 value for some types of TKIs, such as sorafenib, tipifarnib, veliparib, olaparib, which suggested that the DMRegs_score may be predictive of TKIs and ICIs combination therapy for HCC.

In comparison to existing studies of prognostic signatures of HCC, this study has some notable advantages and limitations. First, the global DNA methylation regulators landscape was modeled in order to systematically examine the effects of DNA methylation regulators on TME in HCC patients, which have not been clarified before. Furthermore, we examined the possible role of DNA methylation regulators-related status in predicting the clinical response to immunotherapy in HCC. Our data give rules about how DNA methylation regulators influenced the multiplicity of TME. Secondly, all analyses and samples were obtained primarily based on bioinformatics analysis, and although we did some clinical validation, it is indispensable to conduct prospective studies to further validate the efficacy of DMRegs_score. Besides, a few important clinical variables like surgery and chemoradiotherapy were missing, which could have affected the prognosis of DNA methylation regulators status and the immune response.

In summary, in our comprehensive research on DNA methylation regulators, we uncovered a broad range of regulatory mechanisms in HCC through which they affected clinicopathological features, TME, and prognosis. Additionally, we investigated the therapeutic effects of DNA methylation regulators in targeted therapy and immunotherapy in HCC. Our study emphasized the important clinical implications of DNA methylation regulators and provide new insights into how to personalize immunotherapy for patients with HCC.

## Data availability statement

The original contributions presented in the study are included in the article/**Supplementary Material**. Further inquiries can be directed to the corresponding authors.

## Ethics statement

The studies involving human participants were reviewed and approved by the institutional review committee of the First Affiliated Hospital of Xi’an Jiaotong University, Shaanxi Province, Xi’an, China. The patients/participants provided their written informed consent to participate in this study. Written informed consent was obtained from the individual(s) for the publication of any potentially identifiable images or data included in this article.

## Author contributions

All authors listed have made a substantial, direct, and intellectual contribution to the work, and approved it for publication.

## Funding

This work was supported by the National Natural Science Foundation of China under Granted (number 82102801& number 81902413); and Key Research and Development Program of Shaanxi under Granted number 2019SF-044 & number.2019SF-129.

## Acknowledgments

We would like to thank all teammates for contributing this work.

## Conflict of interest

The authors declare that the research was conducted in the absence of any commercial or financial relationships that could be construed as a potential conflict of interest.

## Publisher’s note

All claims expressed in this article are solely those of the authors and do not necessarily represent those of their affiliated organizations, or those of the publisher, the editors and the reviewers. Any product that may be evaluated in this article, or claim that may be made by its manufacturer, is not guaranteed or endorsed by the publisher.

## Supplementary material

Supplementary Figure 1 The unsupervised clustering of DNA methylation regulators and consensus matrix heatmaps. **(A)** Cumulative distribution function (CDF) curve. **(B)** CDF Delta area curve, which indicates the relative change in the area under the CDF curve for each category number k compared with k-1. **(C)** The consensus matrix heatmaps for k = 2, and k = 4-9.

Supplementary Figure 2 The GSVA analysis of DNA methylaiton regulators-related patterns between pattern **(A–C)** and pattern **(B, C)**.

Supplementary Figure 3 The unsupervised clustering of DNA methylation regulators-related DEGs and consensus matrix heatmaps. **(A)** Venn diagram of DEGs among three DNA methylation regulators-related patterns. **(B)** CDF Delta area curve. **(C)** The consensus matrix heatmaps for k = 2, and k = 4-9.

Supplementary Figure 4 Characteristics of eight genes in the risk model. **(A)** The prognosis of eight genes in HCC patients. **(B–I)** The expression differences of eight genes in two risk groups.

Supplementary Figure 5 Landscape of genetic variations and transcriptional expression of eight genes. **(A)** The expression level of eight genes between different risk groups with clinical features. **(B)** 7 of the 364 patients occurred genetic variations of eight genes with 1.92% mutation frequencies. **(C)** The locations of CNV variations in eight genes on 23 chromosomes. **(D)** The frequency of CNV gain or loss among eight genes.

Supplementary Figure 6 Stratification analysis of the DMRegs_score in HCC. **(A, B)** Age (age ≤ 60 and age > 60 years old). **(C, D)** Gender (female and male). **(E, F)** Tumor stage (I-II or III-IV).

Supplementary Figure 7 The relationship between DMRegs_score and therapeutic sensitivity.

Supplementary Figure 8 The univariate and multivariate cox analysis of DMRegs_score in validating set.

## References

[1] Sung H Ferlay J Siegel RL Laversanne M Soerjomataram I Jemal A. Global cancer statistics 2020: GLOBOCAN estimates of incidence and mortality worldwide for 36 cancers in 185 countries. CA Cancer J Clin (2021) 71(3):209–49. doi: 10.3322/caac.21660 33538338

[2] Llovet JM Kelley RK Villanueva A Singal AG Pikarsky E Roayaie S. Hepatocellular carcinoma. Nat Rev Dis Primers (2021) 7(1):6. doi: 10.1038/s41572-020-00240-3 33479224 PMC13537433

[3] Forner A Reig M Bruix J. Hepatocellular carcinoma. Lancet (2018) 391(10127):1301–14. doi: 10.1016/S0140-6736(18)30010-2 29307467

[4] Li E Zhang Y. DNA Methylation in mammals. Cold Spring Harb Perspect Biol (2014) 6(5):a019133. doi: 10.1101/cshperspect.a019133 24789823 PMC3996472

[5] Smith ZD Meissner A. DNA Methylation: roles in mammalian development. Nat Rev Genet (2013) 14(3):204–20. doi: 10.1038/nrg3354 23400093

[6] Biswas S Rao CM. Epigenetic tools (The writers, the readers and the erasers) and their implications in cancer therapy. Eur J Pharmacol (2018) 837:8–24. doi: 10.1016/j.ejphar.2018.08.021 30125562

[7] Greenberg MVC Bourc'his D. The diverse roles of DNA methylation in mammalian development and disease. Nat Rev Mol Cell Biol (2019) 20(10):590–607. doi: 10.1038/s41580-019-0159-6 31399642

[8] Meng Q Lu YX Ruan DY Yu K Chen YX Xiao M. DNA Methylation regulator-mediated modification patterns and tumor microenvironment characterization in gastric cancer. Mol Ther Nucleic Acids (2021) 24:695–710. doi: 10.1016/j.omtn.2021.03.023 33996253 PMC8099484

[9] Jones PA Ohtani H Chakravarthy A De Carvalho DD. Epigenetic therapy in immune-oncology. Nat Rev Cancer (2019) 19(3):151–61. doi: 10.1038/s41568-019-0109-9 30723290

[10] Dunn J Rao S. Epigenetics and immunotherapy: The current state of play. Mol Immunol (2017) 87:227–39. doi: 10.1016/j.molimm.2017.04.012 28511092

[11] Wei G Zhang H Zhao H Wang J Wu N Li L. Emerging immune checkpoints in the tumor microenvironment: Implications for cancer immunotherapy. Cancer Lett (2021) 511:68–76. doi: 10.1016/j.canlet.2021.04.021 33957184

[12] Herbst RS Baas P Perez-Gracia JL Felip E Kim DW Han JY. Use of archival versus newly collected tumor samples for assessing PD-L1 expression and overall survival: an updated analysis of KEYNOTE-010 trial. Ann Oncol (2019) 30(2):281–9. doi: 10.1093/annonc/mdy545 PMC6931268 30657853

[13] Sacco AG Chen R Worden FP Wong DJL Adkins D Swiecicki P. Pembrolizumab plus cetuximab in patients with recurrent or metastatic head and neck squamous cell carcinoma: an open-label, multi-arm, non-randomised, multicentre, phase 2 trial. Lancet Oncol (2021) 22(6):883–892. doi: 10.1016/S1470-2045(21)00136-4 PMC12140401 33989559

[14] Pires da Silva I Ahmed T Reijers ILM Weppler AM Betof Warner A Patrinely JR. Ipilimumab alone or ipilimumab plus anti-PD-1 therapy in patients with metastatic melanoma resistant to anti-PD-(L)1 monotherapy: a multicentre, retrospective, cohort study. Lancet Oncol (2021) 22(6):836–847. doi: 10.1016/S1470-2045(21)00097-8 33989557

[15] Sangro B Gomez-Martin C de la Mata M Iñarrairaegui M Garralda E Barrera P. A clinical trial of CTLA-4 blockade with tremelimumab in patients with hepatocellular carcinoma and chronic hepatitis c. J Hepatol (2013) 59(1):81–8. doi: 10.1016/j.jhep.2013.02.022 23466307

[16] Yau T Zagonel V Santoro A Acosta-Rivera M Choo SP Matilla A. Nivolumab (NIVO) plus ipilimumab (IPI) plus cabozantinib (CABO) combination therapy in patients (pts) with advanced hepatocellular carcinoma (aHCC): Results from CheckMate 040. J Clin Oncol (2020) 38(4). doi: 10.1200/JCO.2020.38.4_suppl.478

[17] Lee M Ryoo BY Hsu CH Numata K Stein S Verret W. Randomised efficacy and safety results for atezolizumab (Atezo) plus bevacizumab (Bev) in patients (pts) with previously untreated, unresectable hepatocellular carcinoma (HCC). Ann Oncol (2019) 30:875–5. doi: 10.1093/annonc/mdz394.030

[18] Zhu AX Finn RS Edeline J Cattan S Ogasawara S Palmer D. Pembrolizumab in patients with advanced hepatocellular carcinoma previously treated with sorafenib (KEYNOTE-224): a non-randomised, open-label phase 2 trial. Lancet Oncol (2018) 19(7):940–52. doi: 10.1016/S1470-2045(18)30351-6 29875066

[19] Samstein RM Lee CH Shoushtari AN Hellmann MD Shen R Janjigian YY. Tumor mutational load predicts survival after immunotherapy across multiple cancer types. Nat Genet (2019) 51(2):202–6. doi: 10.1038/s41588-018-0312-8 PMC6365097 30643254

[20] Schumacher TN Kesmir C van Buuren MM. Biomarkers in cancer immunotherapy. Cancer Cell (2015) 27(1):12–4. doi: 10.1016/j.ccell.2014.12.004 25584891

[21] Roszik J Haydu LE Hess KR Oba J Joon AY Siroy AE. Novel algorithmic approach predicts tumor mutation load and correlates with immunotherapy clinical outcomes using a defined gene mutation set. BMC Med (2016) 14(1):168. doi: 10.1186/s12916-016-0705-4 27776519 PMC5078889

[22] Jung H Kim HS Kim JY Sun JM Ahn JS Ahn MJ. DNA Methylation loss promotes immune evasion of tumours with high mutation and copy number load. Nat Commun (2019) 10(1):4278. doi: 10.1038/s41467-019-12159-9 31537801 PMC6753140

[23] Zhang B Yang C Wang R Wu J Zhang Y Liu D. OTUD7B suppresses smac mimetic-induced lung cancer cell invasion and migration *via* deubiquitinating TRAF3. J Exp Clin Cancer Res (2020) 39(1):244. doi: 10.1186/s13046-020-01751-3 33198776 PMC7667862

[24] Wilkerson MD Hayes DN. ConsensusClusterPlus: a class discovery tool with confidence assessments and item tracking. Bioinformatics (2010) 26(12):1572–3. doi: 10.1093/bioinformatics/btq170 PMC2881355 20427518

[25] Hänzelmann S Castelo R Guinney J. GSVA: gene set variation analysis for microarray and RNA-seq data. BMC Bioinf (2013) 14:7. doi: 10.1186/1471-2105-14-7 PMC3618321 23323831

[26] Yoshihara K Shahmoradgoli M Martínez E Vegesna R Kim H Torres-Garcia W. Inferring tumour purity and stromal and immune cell admixture from expression data. Nat Commun (2013) 4:2612. doi: 10.1038/ncomms3612 24113773 PMC3826632

[27] Charoentong P Finotello F Angelova M Mayer C Efremova M Rieder D. Pan-cancer immunogenomic analyses reveal genotype-immunophenotype relationships and predictors of response to checkpoint blockade. Cell Rep (2017) 18(1):248–62. doi: 10.1016/j.celrep.2016.12.019 28052254

[28] Mafi S Mansoori B Taeb S Sadeghi H Abbasi R Cho WC. mTOR-mediated regulation of immune responses in cancer and tumor microenvironment. Front Immunol (2021) 12:774103. doi: 10.3389/fimmu.2021.774103 35250965 PMC8894239

[29] Wu HX Chen YX Wang ZX Zhao Q He MM Wang YN. Alteration in TET1 as potential biomarker for immune checkpoint blockade in multiple cancers. J Immunother Cancer (2019) 7(1):264. doi: 10.1186/s40425-019-0737-3 31623662 PMC6798429

[30] Xu YP Lv L Liu Y Smith MD Li WC Tan XM. Tumor suppressor TET2 promotes cancer immunity and immunotherapy efficacy. J Clin Invest (2019) 129(10):4316–31. doi: 10.1172/JCI129317 PMC6763236 31310587

[31] Koch A Joosten SC Feng Z de Ruijter TC Draht MX Melotte V. Analysis of DNA methylation in cancer: location revisited. Nat Rev Clin Oncol (2018) 15(7):459–66. doi: 10.1038/s41571-018-0004-4 29666440

[32] Dawson MA Kouzarides T. Cancer epigenetics: from mechanism to therapy. Cell (2012) 150(1):12–27. doi: 10.1016/j.cell.2012.06.013 22770212

[33] Wang J Yang J Li D Li J. Technologies for targeting DNA methylation modifications: Basic mechanism and potential application in cancer. Biochim Biophys Acta Rev Cancer (2021) 1875(1):188454. doi: 10.1016/j.bbcan.2020.188454 33075468

[34] Jones PA Issa JP Baylin S. Targeting the cancer epigenome for therapy. Nat Rev Genet (2016) 17(10):630–41. doi: 10.1038/nrg.2016.93 27629931

[35] Hogg SJ Beavis PA Dawson MA Johnstone RW. Targeting the epigenetic regulation of antitumour immunity. Nat Rev Drug Discovery (2020) 19(11):776–800. doi: 10.1038/s41573-020-0077-5 32929243

[36] Pan W Zhu S Qu K Meeth K Cheng J He K. The DNA methylcytosine dioxygenase Tet2 sustains immunosuppressive function of tumor-infiltrating myeloid cells to promote melanoma progression. Immunity (2017) 47(2):284–297.e5. doi: 10.1016/j.immuni.2017.07.020 28813659 PMC5710009

[37] Li S Feng J Wu F Cai J Zhang X Wang H. TET2 promotes anti-tumor immunity by governing G-MDSCs and CD8(+) T-cell numbers. EMBO Rep (2020) 21(10):e49425. doi: 10.15252/embr.201949425 32929842 PMC7534639

[38] Zhang B Wu Q Li B Wang D Wang L Zhou YL. m(6)A regulator-mediated methylation modification patterns and tumor microenvironment infiltration characterization in gastric cancer. Mol Cancer (2020) 19(1):53. doi: 10.1186/s12943-020-01170-0 32164750 PMC7066851

[39] Klümper N Ralser DJ Bawden EG Landsberg J Zarbl R Kristiansen G. LAG3 (LAG-3, CD223) DNA methylation correlates with LAG3 expression by tumor and immune cells, immune cell infiltration, and overall survival in clear cell renal cell carcinoma. J Immunother Cancer (2020) 8(1):e000552. doi: 10.1136/jitc-2020-000552 32234847 PMC7174079

[40] Zheng Y Wang Z Wei S Liu Z Chen G. Epigenetic silencing of chemokine CCL2 represses macrophage infiltration to potentiate tumor development in small cell lung cancer. Cancer Lett (2021) 499:148–63. doi: 10.1016/j.canlet.2020.11.034 33253790

[41] Fu J Zhang Z Zhou L Qi Z Xing S Lv J. Impairment of CD4+ cytotoxic T cells predicts poor survival and high recurrence rates in patients with hepatocellular carcinoma. Hepatology (2013) 58(1):139–49. doi: 10.1002/hep.26054 22961630

[42] Zhu J Feng A Sun J Jiang Z Zhang G Wang K. Increased CD4(+) CD69(+) CD25(-) T cells in patients with hepatocellular carcinoma are associated with tumor progression. J Gastroenterol Hepatol (2011) 26(10):1519–26. doi: 10.1111/j.1440-1746.2011.06765.x 21557772

[43] Lurje I Hammerich L Tacke F. Dendritic cell and T cell crosstalk in liver fibrogenesis and hepatocarcinogenesis: Implications for prevention and therapy of liver cancer. Int J Mol Sci (2020) 21(19):7378. doi: 10.3390/ijms21197378 PMC7583774 33036244

[44] Zongyi Y Xiaowu L. Immunotherapy for hepatocellular carcinoma. Cancer Lett (2020) 470:8–17. doi: 10.1016/j.canlet.2019.12.002 31811905

[45] Kaymak I Williams KS Cantor JR Jones RG. Immunometabolic interplay in the tumor microenvironment. Cancer Cell (2021) 39(1):28–37. doi: 10.1016/j.ccell.2020.09.004 33125860 PMC7837268

[46] Cheng AL Hsu C Chan SL Choo SP Kudo M. Challenges of combination therapy with immune checkpoint inhibitors for hepatocellular carcinoma. J Hepatol (2020) 72(2):307–19. doi: 10.1016/j.jhep.2019.09.025 31954494

[47] Dong Y Wong JSL Sugimura R Lam KO Li B Kwok GGW. Recent advances and future prospects in immune checkpoint (ICI)-based combination therapy for advanced HCC. Cancers (Basel) (2021) 13(8):1949. doi: 10.3390/cancers13081949 33919570 PMC8072916

[48] Tang B Zhu J Zhao Z Lu C Liu S Fang S. Diagnosis and prognosis models for hepatocellular carcinoma patient's management based on tumor mutation burden. J Adv Res (2021) 33:153–65. doi: 10.1016/j.jare.2021.01.018 PMC8463909 34603786

[49] Hong WF Gu YJ Wang N Xia J Zhou HY Zhan K. Integrative characterization of immune-relevant genes in hepatocellular carcinoma. J Clin Transl Hepatol (2021) 9(3):301–14. doi: 10.14218/JCTH.2020.00132 PMC8237144 34221916

