# Supplementary material for: DNA methylation regulators-related molecular patterns and tumor immune landscape in hepatocellular carcinoma
Source: Front Oncol. 2022 Aug 26;12:877817. doi: 10.3389/fonc.2022.877817 (PMC9459088; doi:10.3389/fonc.2022.877817)
Supplement: Supplementary Figure 1 — The unsupervised clustering of DNA methylation regulators and consensus matrix heatmaps. (A) Cumulative distribution function (CDF) curve. (B) CDF Delta area curve, which indicates the relative change in the area under the CDF curve for each category number k compared with k-1. (C) The consensus matrix heatmaps for k = 2, and k = 4-9. [file Presentation_1.pdf]

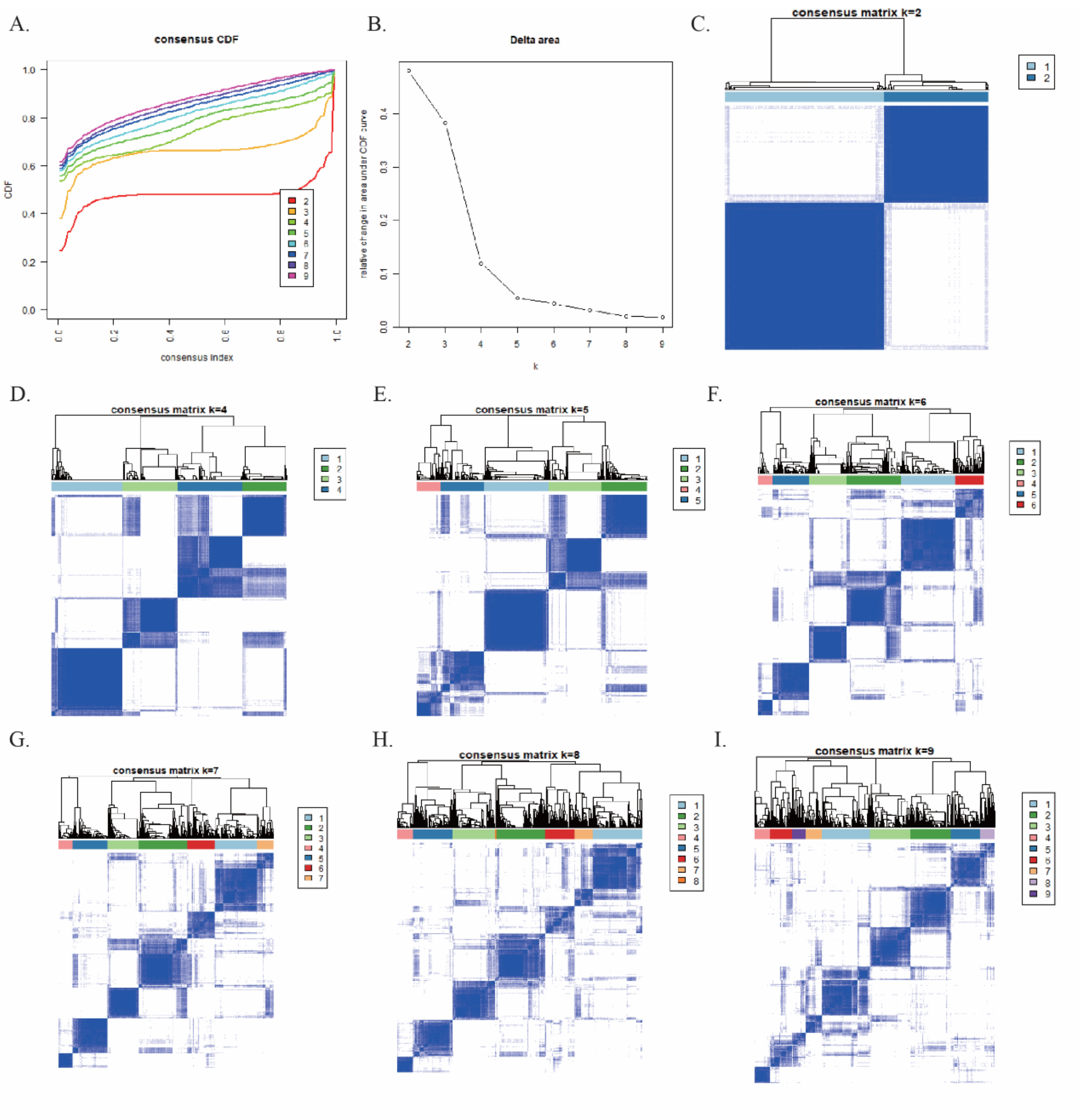

Figure S1. The unsupervised clustering of DNA methylation regulators and consensus matrix heatmaps. (A) Cumulative distribution function (CDF) curve. (B) CDF Delta area curve, which indicates the relative change in the area under the CDF curve for each category number k compared with k-1. (C) The consensus matrix heatmaps for k = 2, and k= 4-9.

Figure S2

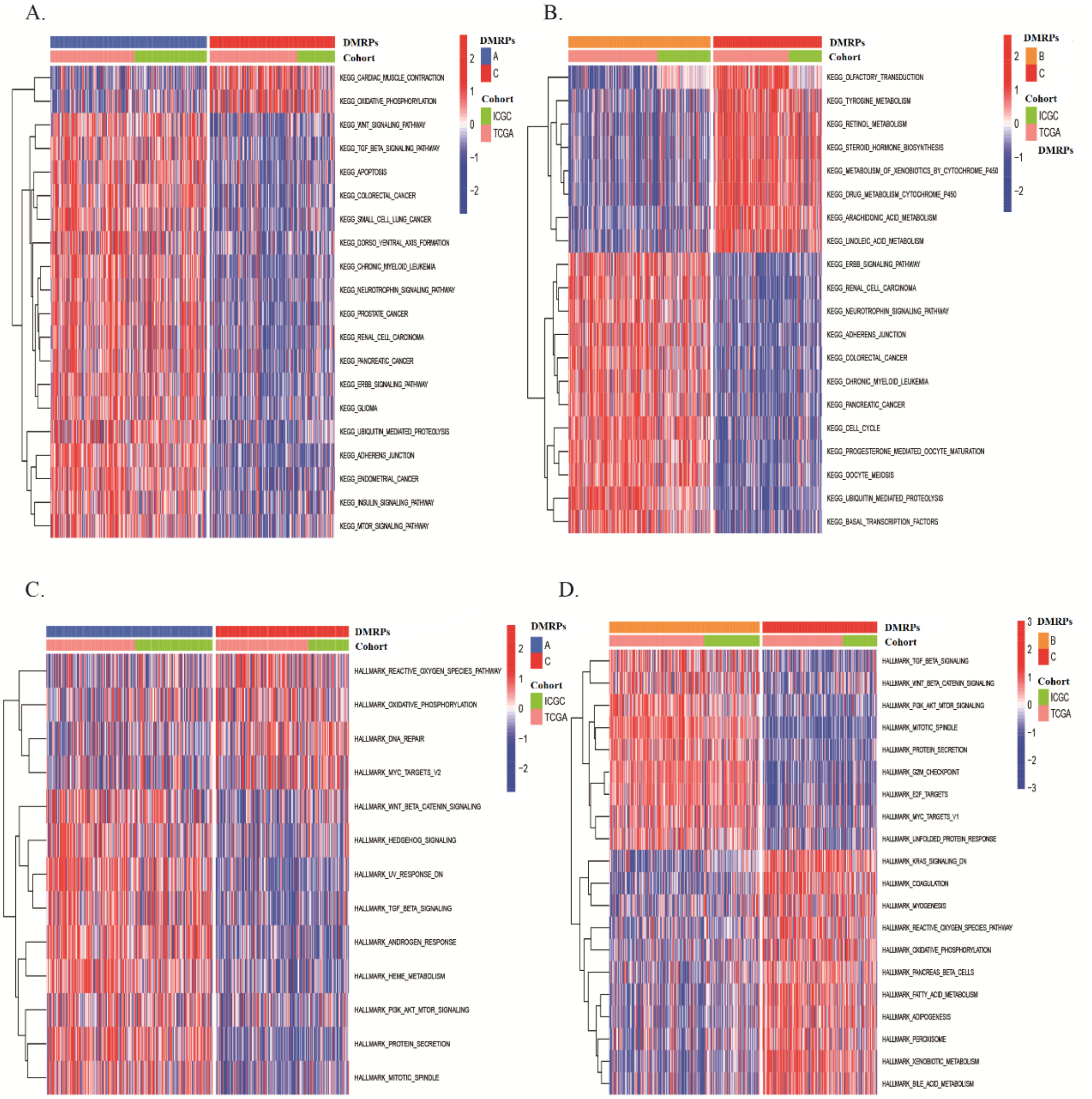

Figure S2. The GSVA analysis of DNA methylaiton regulators-related patterns between pattern A-C and pattern B-C.

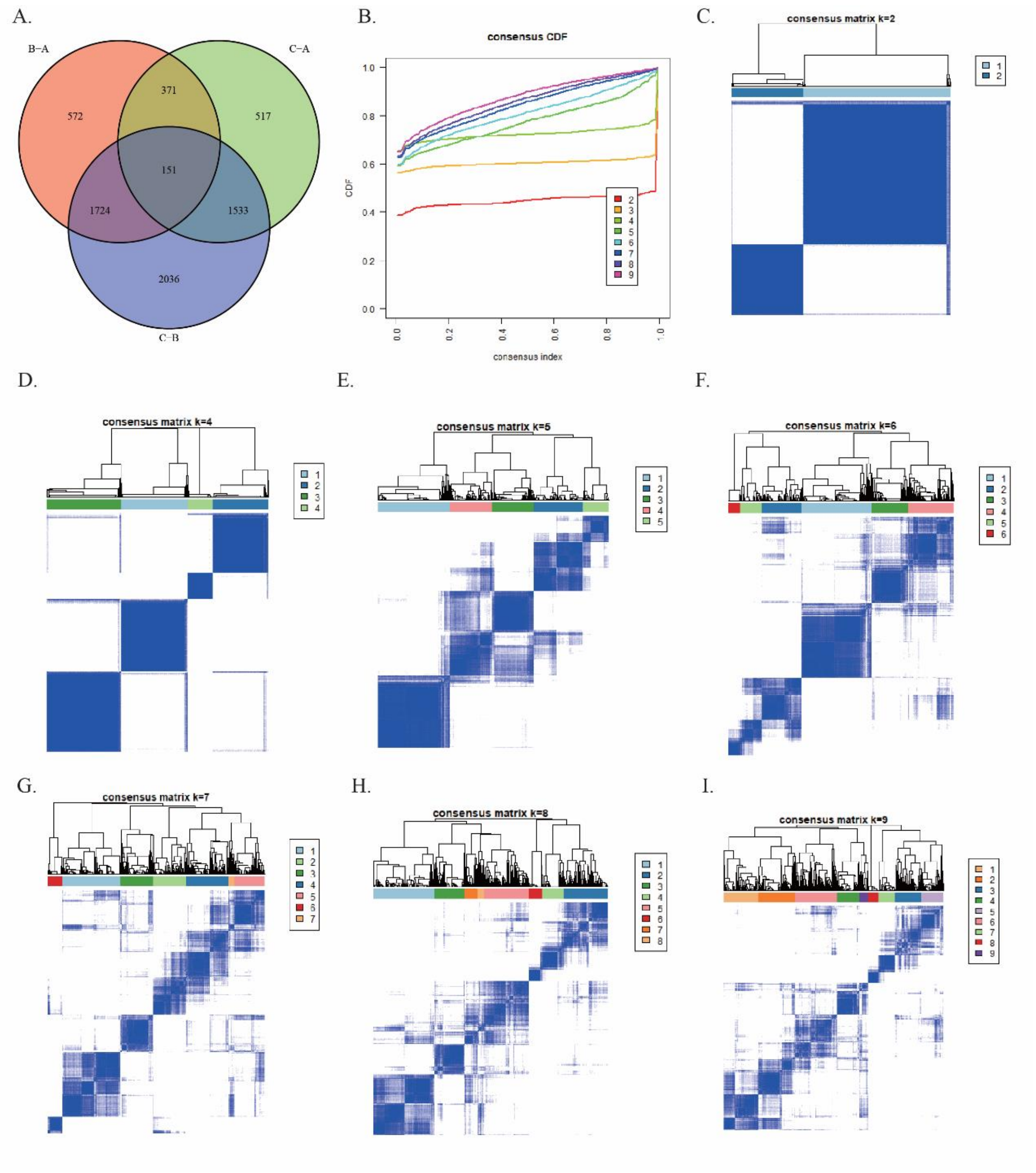

Figure S3. The unsupervised clustering of DNA methylation regulators-related DEGs and consensus matrix heatmaps. (A) Venn diagram of DEGs among three DNA methylation regulators-related patterns. (B) CDF Delta area curve. (C) The consensus matrix heatmaps for k = 2, and k= 4-9.

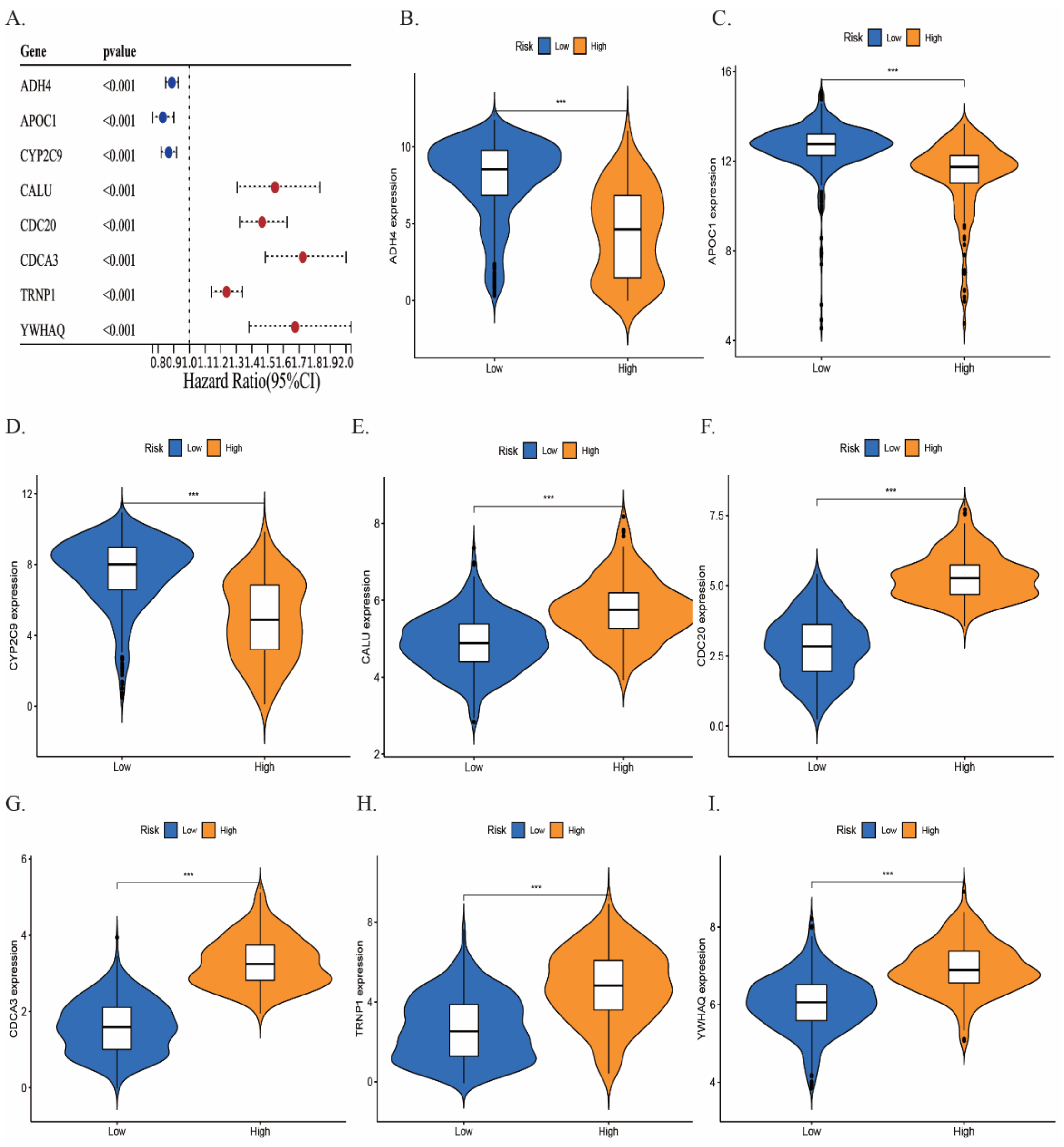

Figure S4. Characteristics of eight genes in the risk model. (A) The prognosis of eight genes in HCC patients. (B-I) The expression differences of eight genes in two risk groups.

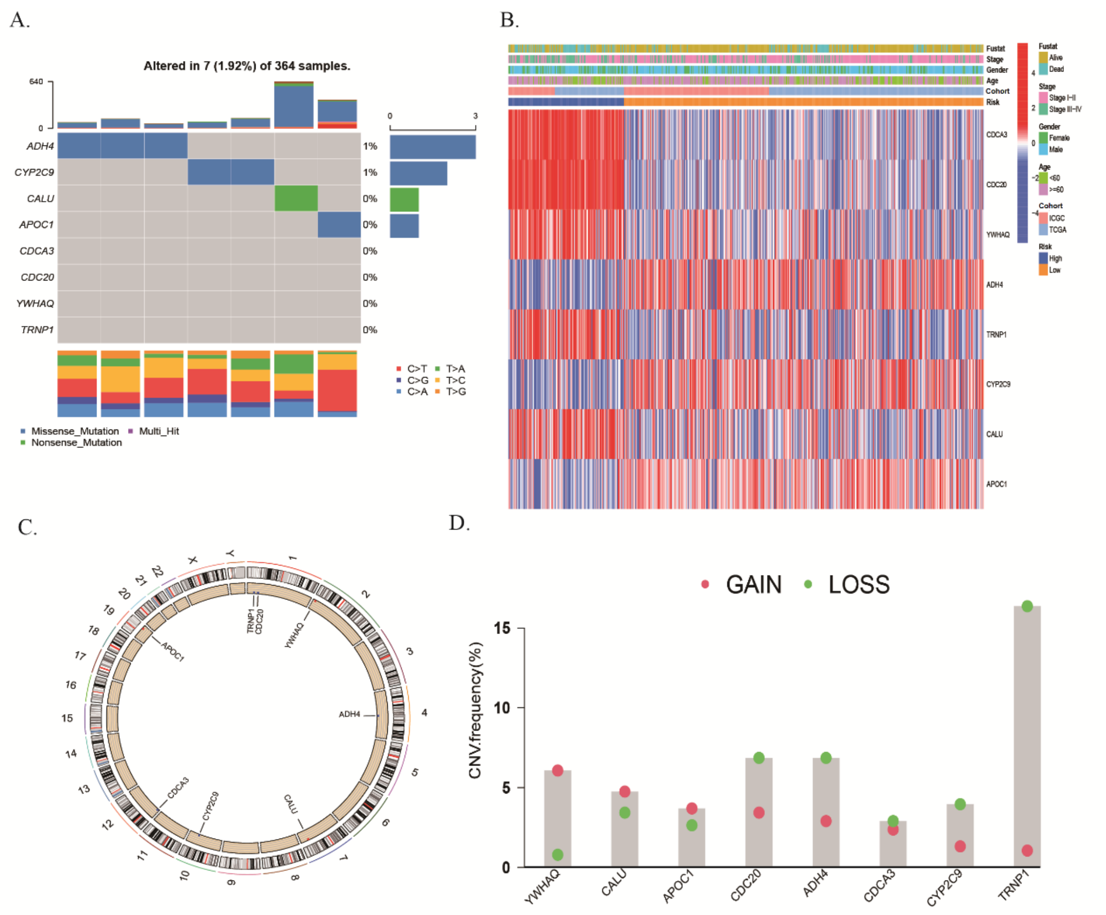

Figure S5. Landscape of genetic variations and transcriptional expression of eight genes. (A) The expression level of eight genes between different risk groups with clinical features. (B) 7 of the 364 patients occurred genetic variations of eight genes with 1.92% mutation frequencies. (C) The locations of CNV variations in eight genes on 23 chromosomes. (D) The frequency of CNV gain or loss among eight genes.

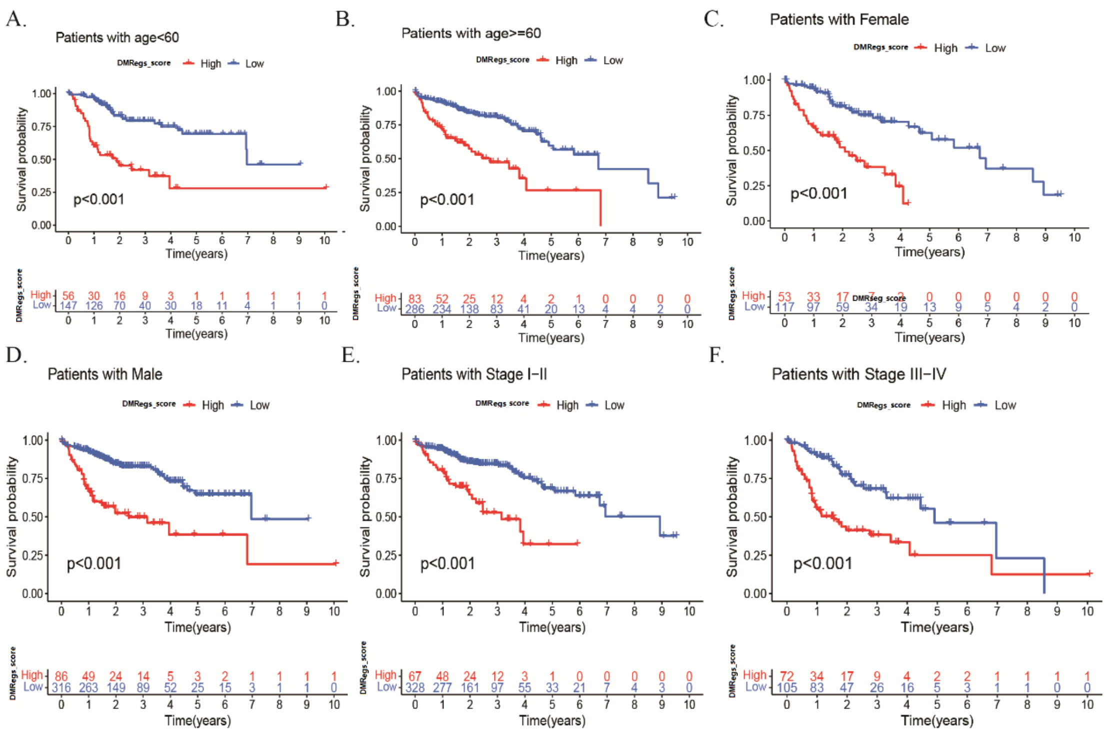

Figure S6. Stratification analysis of the DMRegs_ score in HCC. (A-B) Age (age ≤ 60 and age > 60 years old). (C-D) Gender (female and male). (E-F) Tumor stage (I-II or III-IV).

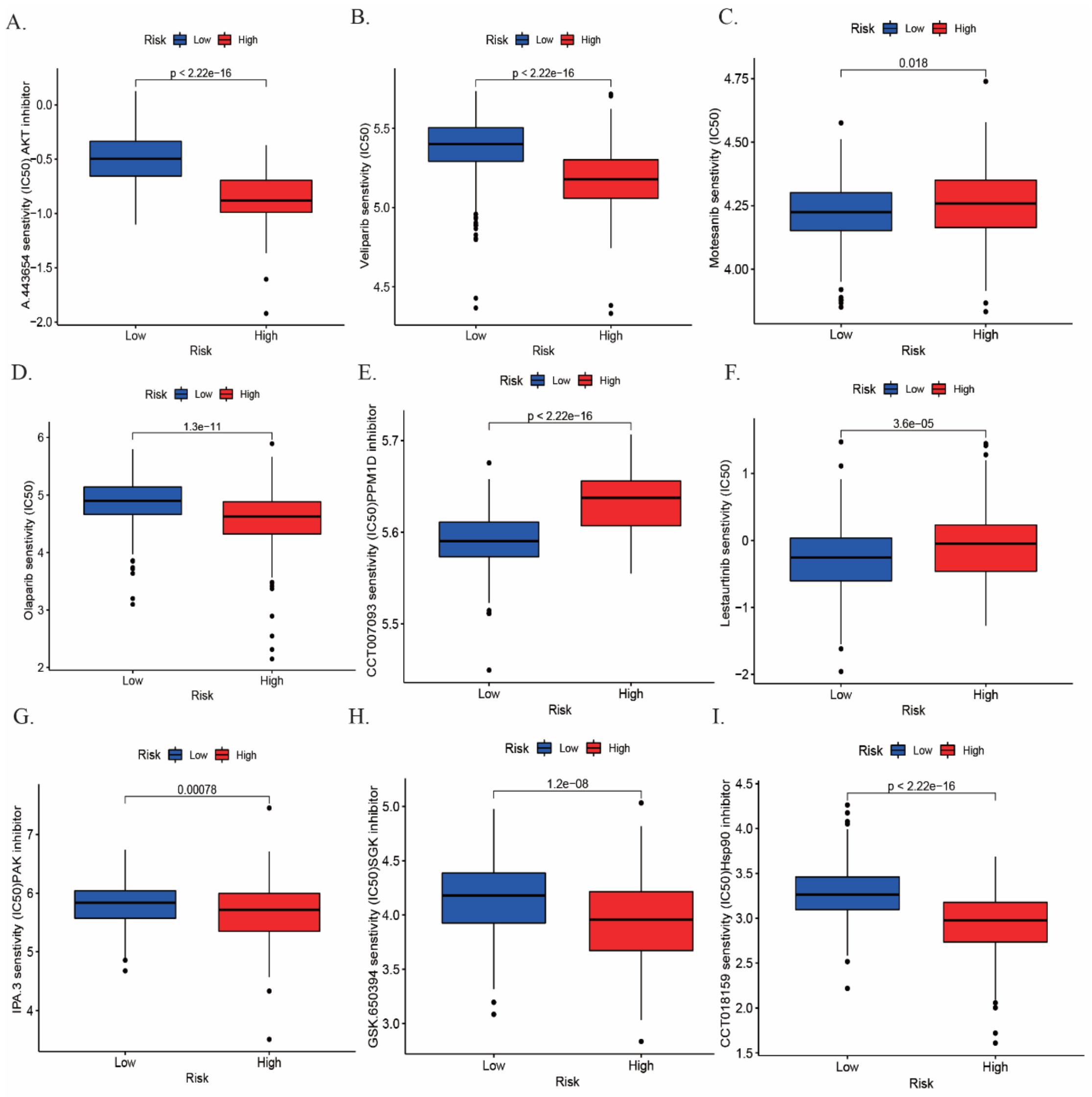

Figure S7. The relationship between DMRegs_score and therapeutic sensitivity.

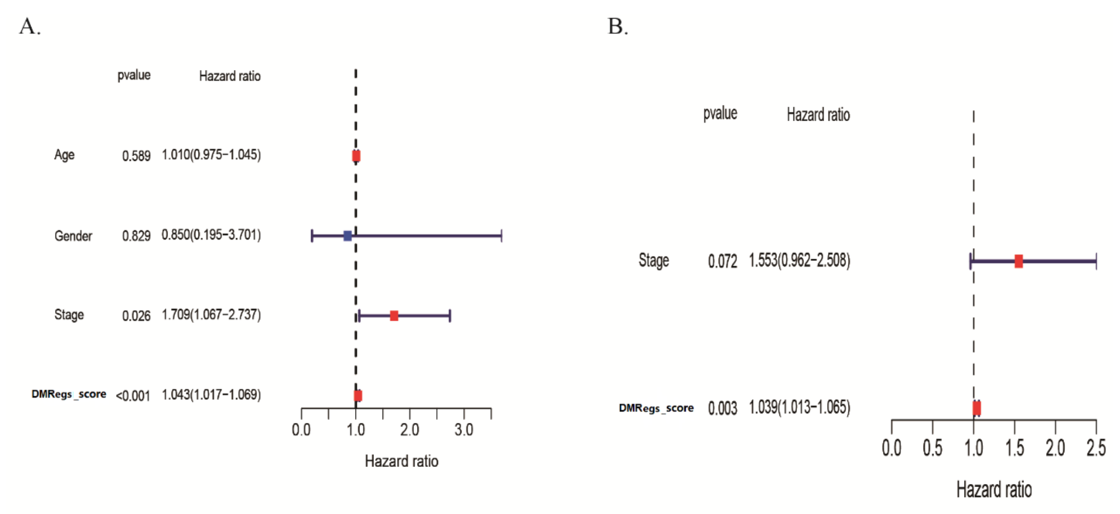

Figure S8. The univariate and multivariate cox analysis of DMRegs_score in validating set.
