# Supplementary material for: DNA methylation regulators-related molecular patterns and tumor immune landscape in hepatocellular carcinoma
Source: Front Oncol. 2022 Aug 26;12:877817. doi: 10.3389/fonc.2022.877817 (PMC9459088; doi:10.3389/fonc.2022.877817)
Supplement: Supplementary file 4 [file DataSheet_3.docx]

R script

Transform FPKM to TPM

library(limma)

inputFile="symbol.txt"

setwd("D:\\sunny\\Bioinformation\\4.DNA m6A\\1.TPM")

outTab=data.frame()

rt=read.table(inputFile, header=T, sep="\t", check.names=F)

rt=as.matrix(rt)

rownames(rt)=rt[,1]

exp=rt[,2:ncol(rt)]

dimnames=list(rownames(exp),colnames(exp))

data=matrix(as.numeric(as.matrix(exp)),nrow=nrow(exp),dimnames=dimnames)

data=avereps(data)

fpkmToTpm=function(fpkm){

exp(log(fpkm) - log(sum(fpkm)) + log(1e6))

}

tpm=apply(data, 2, fpkmToTpm)

tpmOut=rbind(ID=colnames(tpm), tpm)

write.table(tpmOut, file="TCGA.TPM.txt", sep="\t", col.names=F, quote=F)

Diffrentially expressed genes analysis

library(limma)

library(reshape2)

library(ggpubr)

expFile="TCGA.TPM.txt"

geneFile="gene.txt"

setwd("D:\\sunny\\Bioinformation\\4.DNA m6A\\2.diff")

rt=read.table(expFile, header=T, sep="\t", check.names=F)

rt=as.matrix(rt)

rownames(rt)=rt[,1]

exp=rt[,2:ncol(rt)]

dimnames=list(rownames(exp), colnames(exp))

data=matrix(as.numeric(as.matrix(exp)), nrow=nrow(exp), dimnames=dimnames)

data=avereps(data)

gene=read.table(geneFile, header=T, sep="\t", check.names=F)

sameGene=intersect(as.vector(gene[,1]), row.names(data))

data=data[sameGene,]

group=sapply(strsplit(colnames(data),"\\-"), "[", 4)

group=sapply(strsplit(group,""), "[", 1)

group=gsub("2", "1", group)

conNum=length(group[group==1])

treatNum=length(group[group==0])

sampleType=c(rep(1,conNum), rep(2,treatNum))

exp=log2(data+1)

exp=as.data.frame(t(exp))

exp=cbind(exp, Type=sampleType)

exp$Type=ifelse(exp$Type==1, "Normal", "Tumor")

data=melt(exp, id.vars=c("Type"))

colnames(data)=c("Type", "Gene", "Expression")

p=ggboxplot(data, x="Gene", y="Expression", color = "Type",

ylab="Gene expression",

xlab="",

legend.title="Type",

palette = c("#7CC767","#D20A13"),

width=1)

p=p+rotate_x_text(60)

p1=p+stat_compare_means(aes(group=Type),

method="wilcox.test",

symnum.args=list(cutpoints = c(0, 0.001, 0.01, 0.05, 1), symbols = c("***", "**", "*", " ns")),

label = "p.signif")

pdf(file="boxplot5.pdf", width=7, height=5)

print(p1)

dev.off()
